# Supplementary material for: Functional and structural insights into the multi-step activation and catalytic mechanism of bacterial ExoY nucleotidyl cyclase toxins bound to actin-profilin
Source: PLoS Pathog. 2023 Sep 25;19(9):e1011654. doi: 10.1371/journal.ppat.1011654 (PMC10553838; doi:10.1371/journal.ppat.1011654)
Supplement: S1 Table — Compared to the Kd of ~0.1–60 nM for CyaA or EF binding to CaM [34,35] or of ~1 μM for Pa-ExoY binding to F-actin [3], Vn-ExoY exhibits only a modest affinity for G-actin (Fig 1D and 1E, Kd~12 μM). The modest affinity of Vn-ExoY is balanced by its ability to interact with and efficiently activate G-actin bound to Profilin (Fig 2A–2C), since the G-actin:Profilin complex is abundant in eukaryotic cells [36,39]. Km, Vmax and Kcat values for different nucleotide substrates and potential NC toxin inhibitors have only been characterized for the calmodulin-activated NC toxins EF and CyaA. The references given are: (Wolff et al. 1980) [91], (Shen et al. 2002) [34], (Gottle et al. 2010) [43], (Soelaiman et al. 2003) [92], (Leppla 1982) [93], (Laine et al. 2010) [94], (Lee et al. 2004) [95], (Belyy et al. 2016) [3], (Becker et al. 2014) [16], (Raoux-Barbot et al. 2018) [5], (Stein, 2022) [35], and (Drum et al., 2000) [96]. (DOCX) [file ppat.1011654.s001.docx]

| **NC toxin** | **Cofactor**  **(K_D_ for cofactor binding)** | **Substrate specificity or increased cNMP levels in cells infected by P. a. strains** | **Kcat (s^-1^)** | **Km (µM)** | **Inhibitors** |
| --- | --- | --- | --- | --- | --- |
| **CyaA** | ●**Calmodulin** [1]  K_D_ **~ 0.2 nM** [2]  K_D_ **~ 60 / 0.06 nM** [3] | **ATP >> CTP** [4] | ● AC: 332.2 ± 42.4 (Mn^2+^)  ● CC: 8.9 ± 2.0 (Mn^2+^)  ● UC: 0.3 ± 0.05 (Mn^2+^)  [4]  ● AC: 1000±125 / 500±50 (Mg^2+^) [3] | ● AC: 57.7 ± 4.6 (Mn^2+^)  ● CC: 46.6 ± 9.7 (Mn^2+^)  ● UC: 68.6 ± 9.8 (Mn^2+^)  [4]  ● AC: ~300 (Mg^2+^) [3] | ●Adefovir diphosphate  Ki= 25 nM [5] |
| **EF** | ●**Calmodulin** [6]  K_D_ **~ 20 nM** [2]  ≥1,000-fold stimulation of its basal AC activity by CaM [7] | **ATP >> CTP** [4] | ● AC: 684.2 ± 272.5 (Mg^2+^)  ● CC: 7.2 ± 3.1 (Mg^2+^)  ● AC: 501.5 ± 55.9 (Mn^2+^)  ● CC: 8.8 ± 1.4 (Mn^2+^)  ● UC: 2.3 ± 0.2 (Mn^2+^)  [4] | ● AC: 175.8 ± 29.9 (Mg^2+^)  ● CC: 419.7 ± 115.1 (Mg^2+^)  ● AC: 35.3 ± 3.7 (Mn^2+^)  ● CC: 12.5 ± 3.4 (Mn^2+^)  ● UC: 134.5 ± 23.5 (Mn^2+^)  [4] | ●Adefovir diphosphate  Ki= 27 nM *in vitro* [5, 8]  ●TUAdiCl 3,4-diCl  (thiophen ureidoacids scaffold)  IC50=2µM [9],  ●4-[4-(4-nitrophenyl)-thiazolylamino]-benzene-sulfonamide  IC50=10µM [10] |
| **Pa-ExoY** | ●**F-actin**  K_D_ **~ 1 µM**  >10,000-fold stimulation of its basal GC or AC activity by F-actin [11] | ●in infected B103 cells:  **cUMP~cGMP>cAMP>cCMP**  ●in infected A549 / HeLa cells:  **cUMP>cGMP>cCMP>cAMP** [12]  ●*in vitro* with  Mg^2+^: **GTP>ATP≥UTP>CTP**  Mn^2+^: **ATP~GTP>UTP~CTP** [13] | ●GC: 1000 (Mg^2+^) [11] |  | unknown |
| **Vn-ExoY** | ●**G-actin** [13]  ●**G-actin:profilin**  K_D_ **~ 12 µM**  >30,000-fold stimulation of its basal AC activity by G-actin:profilin [current work] | *in vitro* with Mg^2+^ or Mn^2+^:  **ATP>> CTP>UTP** [13] |  |  | unknown |

**S1 Table.**

**References**

1. Wolff J, Cook GH, Goldhammer AR, Berkowitz SA. Calmodulin activates prokaryotic adenylate cyclase. Proc Natl Acad Sci U S A. 1980;77(7):3841-4. doi: 10.1073/pnas.77.7.3841. PubMed PMID: 6253992; PubMed Central PMCID: PMCPMC349722.

2. Shen Y, Lee YS, Soelaiman S, Bergson P, Lu D, Chen A, et al. Physiological calcium concentrations regulate calmodulin binding and catalysis of adenylyl cyclase exotoxins. EMBO J. 2002;21(24):6721-32. doi: 10.1093/emboj/cdf681. PubMed PMID: 12485993; PubMed Central PMCID: PMCPMC139101.

3. Stein RL. Kinetic Studies of the Activation of Bordetella pertussis Adenylate Cyclase by Calmodulin. Biochemistry. 2022;61(7):554-62. doi: 10.1021/acs.biochem.1c00824. PubMed PMID: 35263092.

4. Gottle M, Dove S, Kees F, Schlossmann J, Geduhn J, Konig B, et al. Cytidylyl and uridylyl cyclase activity of bacillus anthracis edema factor and Bordetella pertussis CyaA. Biochemistry. 2010;49(26):5494-503. PubMed PMID: 20521845.

5. Shen Y, Zhukovskaya NL, Zimmer MI, Soelaiman S, Bergson P, Wang CR, et al. Selective inhibition of anthrax edema factor by adefovir, a drug for chronic hepatitis B virus infection. Proc Natl Acad Sci U S A. 2004;101(9):3242-7. doi: 10.1073/pnas.0306552101. PubMed PMID: 14978283; PubMed Central PMCID: PMCPMC365774.

6. Leppla SH. Anthrax toxin edema factor: a bacterial adenylate cyclase that increases cyclic AMP concentrations of eukaryotic cells. Proc Natl Acad Sci U S A. 1982;79(10):3162-6. PubMed PMID: 6285339.

7. Drum CL, Yan SZ, Sarac R, Mabuchi Y, Beckingham K, Bohm A, et al. An extended conformation of calmodulin induces interactions between the structural domains of adenylyl cyclase from Bacillus anthracis to promote catalysis. J Biol Chem. 2000;275(46):36334-40. doi: 10.1074/jbc.M004778200. PubMed PMID: 10926933.

8. Soelaiman S, Wei BQ, Bergson P, Lee YS, Shen Y, Mrksich M, et al. Structure-based inhibitor discovery against adenylyl cyclase toxins from pathogenic bacteria that cause anthrax and whooping cough. J Biol Chem. 2003;278(28):25990-7. doi: 10.1074/jbc.M301232200. PubMed PMID: 12676933.

9. Laine E, Goncalves C, Karst JC, Lesnard A, Rault S, Tang WJ, et al. Use of allostery to identify inhibitors of calmodulin-induced activation of Bacillus anthracis edema factor. Proc Natl Acad Sci U S A. 2010;107(25):11277-82. doi: 10.1073/pnas.0914611107. PubMed PMID: 20534570; PubMed Central PMCID: PMCPMC2895076.

10. Lee YS, Bergson P, He WS, Mrksich M, Tang WJ. Discovery of a small molecule that inhibits the interaction of anthrax edema factor with its cellular activator, calmodulin. Chem Biol. 2004;11(8):1139-46. doi: 10.1016/j.chembiol.2004.05.020. PubMed PMID: 15324815.

11. Belyy A, Raoux-Barbot D, Saveanu C, Namane A, Ogryzko V, Worpenberg L, et al. Actin activates Pseudomonas aeruginosa ExoY nucleotidyl cyclase toxin and ExoY-like effector domains from MARTX toxins. Nat Commun. 2016;7:13582. doi: 10.1038/ncomms13582. PubMed PMID: 27917880; PubMed Central PMCID: PMCPMC5150216.

12. Beckert U, Wolter S, Hartwig C, Bahre H, Kaever V, Ladant D, et al. ExoY from Pseudomonas aeruginosa is a nucleotidyl cyclase with preference for cGMP and cUMP formation. Biochem Biophys Res Commun. 2014;450(1):870-4. PubMed PMID: 24971548.

13. Raoux-Barbot D, Belyy A, Worpenberg L, Montluc S, Deville C, Henriot V, et al. Differential regulation of actin-activated nucleotidyl cyclase virulence factors by filamentous and globular actin. PLoS One. 2018;13(11):e0206133. doi: 10.1371/journal.pone.0206133. PubMed PMID: 30419035; PubMed Central PMCID: PMCPMC6231621.
