# Supplementary material for: Functional and structural insights into the multi-step activation and catalytic mechanism of bacterial ExoY nucleotidyl cyclase toxins bound to actin-profilin
Source: PLoS Pathog. 2023 Sep 25;19(9):e1011654. doi: 10.1371/journal.ppat.1011654 (PMC10553838; doi:10.1371/journal.ppat.1011654)
Supplement: S2 Table — The first and last residues indicated in the Vn-ExoY constructs correspond to those of the Uniprot sequence A0A6N3LUE9_9VIBR from the MARTX toxin of Vibrio nigripulchritudo, retaining the full numbering. In the text and figures, however, the thousands, i.e. 3000, have been omitted for ease of reading. (DOCX) [file ppat.1011654.s002.docx]

| **Complex** | **1** | | **2** | | **3** | **4** | **5** | **6** |
| --- | --- | --- | --- | --- | --- | --- | --- | --- |
| **Complex** | **Vn-ExoY**  **(residue**^#^ **Q3455 to P3896 with K3528M, K3535I)**  **fused to PRM-profilin: ADP-actin-LatB** | | **SO_4_^2-^-**  **bound**  **Vn-ExoY**  **(residue**  **Q3455 to P3896)**  **fused to PRM-profilin:**  **ADP-actin** | | **SO_4_^2-^-**  **bound**  **Vn-ExoY (wt)**  **(residue Q3455 to L3863):**  **ATP-actin-LatB:**  **profilin** | **(3'dATP-2Mg^2+^)-**  **bound Vn-ExoY (wt)**  **(residue**^#^ **Q3455 to L3863):**  **ATP-actin-LatB** | **(3'dATP-2(Mn/Mg)^2+^)-bound Vn-ExoY(wt)**  **(residue Q3455 to L3863):**  **ATP-actin-LatB** | **(3'dCTP-2(Mn)^2+^)-bound Vn-ExoY(wt)**  **(residue Q3455 to L3863):**  **ATP-actin-LatB** |
| **PDB code** | **8BJH** | | **8BJI** | | **8BJJ** | **8BR1** | **8BO1** | **8BR0** |
| Synchrotron / beamline | SOLEIL / PROXIMA-1 | | SOLEIL / PROXIMA-1 | | SOLEIL / PROXIMA-2 | SOLEIL / PROXIMA-1 | SOLEIL / PROXIMA-2 | SOLEIL / PROXIMA-1 |
| Date of data collection | 12.06.2020 | | 03.07.2020 | | 06.12.2020 | 26.03.2021 | 26.03.2021 | 22.04.2022 |
| Wavelength (Å) | 0.979 | | 0.979 | | 0.980 | 0.979 | 1.893 | 0.979 |
| Resolution range (Å)in data reduction | 93.57–1.69 (1.85–1.69) | | 61.81–1.64 (1.82–1.64) | | 44.20–1.70 (1.73–1.70) | 47.76-2.04 (2.21-2.04) | 48.24-2.50 (2.70-2.50) | 48.05-2.22 (2.36-2.22) |
| Space group | *P2_1_* | | *P2_1_* | | *P2_1_* | *P2_1_* | *P2_1_* | *P2_1_* |
| *a*, *b*, *c* (Å) | 81.74, 62.82, 93.58 | | 81.97, 62.85, 93.59 | | 81.19, 63.20, 93.46 | 76.12, 132.44, 96.40 | 75.50, 132.17, 96.03 | 74.47, 132.50, 96.22 |
| α, β, γ (°) | 90, 91.03, 90 | | 90, 90.28, 90 | | 90, 91.04, 90 | 90, 110.57, 90 | 90, 110.80, 90 | 90, 110.40, 90 |
| Total reflections | 557546 (26053) | | 584789 (29619) | | 1584617 (71381) | 2829414 (138734) | 1287849 (68902) | 518884 (25532) |
| Unique reflections | 81375 (4069) | | 83837 (4192) | | 104207 (5211) | 86495 (4319) | 45059 (2239) | 62059 (2919) |
| Multiplicity | 6.9 (6.4) | | 7.0 (7.1) | | 15.2 (13.7) | 32.7 (32.1) | 28.6 (30.8) | 8.4 (8.7) |
| Completeness spherical (%) | 76.5 (16.3) | | 72.2 (13.9) | | 99.6 (95.3) | 76.6 (18.8) | 74.1 (18.0) | 71.8 (13.7) |
| Completeness ellipsoidal (%) | 94.9 (60.1) | | 94.6 (65.1) | | 99.8 (99.0) | 84.3 (32.1) | 93.7 (57.5) | 92.2 (49.5) |
| Mean *I*/σ(*I*) | 11.2 (1.7) | | 8.5 (1.6) | | 25.3 (2.7) | 14.1 (1.4) | 16.2 (1.0) | 11.8 (1.5) |
| Wilson *B* factor (Å^2^) | 26.5 | | 22.6 | | 24.1 | 51.1 | 75.8 | 64.2 |
| Matthews coefficient (*V*_M_) (Å^3^Da^−1^) | 2.24 | | 2.25 | | 2.36 | 2.58 | 2.54 | 2.63 |
| Solvent content (%) | 45.18 | | 45.36 | | 47.88 | 52.35 | 51.61 | 53.18 |
| *R*_merge_  (all I+ & I-) | 0.086 (0.910) | | 0.123 (1.094) | | 0.057 (1.055) | 0.162 (3.852) | 0.154 (4.021) | 0.098 (1.337) |
| *R*_meas_  (all I+ & I-) | 0.093 (0.837) | | 0.133 (1.182) | | 0.059 (1.095) | 0.164 (3.914) | 0.157 (4.087) | 0.105 (1.420) |
| *R*_p.i.m._  (all I+ & I-) | 0.035 (0.384) | | 0.051 (0.444) | | 0.015 (0.289) | 0.029 (0.688) | 0.029 (0.734) | 0.036 (0.471) |
| CC_1/2_ | 0.998 (0.725) | | 0.996 (0.708) | | 0.999 (0.86) | 0.999 (0.615) | 1.000 (0.575) | 0.998 (0.587) |
| Resolution range (Å) in refinement | 93.57–1.69 (1.80–1.69) | | 25.06-1.75 (1.80-1.75) | | 44.20–1.70 (1.71–1.70) | 47.76-2.04 (2.15-2.04) | 48.24-2.50 (2.63-2.50) | 48.05- 2.22  (2.36-2.22) |
| Reflections used in refinement | 81375 (1539) | | 82105 (1573) | | 104207 (1988) | 86494 (1654) | 45059 (863) | 62059 (1181) |
| Reflections used for *R*_free_ | 4009 (89) | | 4141 (70) | | 5186 (97) | 4302 (76) | 2243 (39) | 2974 (61) |
| *R*_work_ | 0.178 (0.221) | | 0.175 (0.225) | | 0.175 (0.225) | 0.190 (0.215) | 0.191 (0.235) | 0.208 (0.269) |
| *R*_free_ | 0.212 (0.229) | | 0.212 (0.269) | | 0.195 (0.238) | 0.222 (0.240) | 0.231 (0.283) | 0.243 (0.375) |
|  |  |  | |  |  |  |  |  |
| Total nb of atoms | 7980 | | 7926 | | 7763 | 12811 | 12368 | 12 638 |
| Macromolecules | 6900 | | 6857 | | 6922 | 11963 | 11927 | 11927 |
| Ligands | 55 | | 33 | | 64 | 182 | 182 | 170 |
| Solvent | 1025 | | 1036 | | 777 | 666 | 259 | 541 |
| No. of protein residues (per chain) | 366(A),  502(B) | | 366 (A),  498 (B) | | 367 (A),  355 (B),  139 (C) | 358, 365  (A, C),  398, 397  (B, D) | 360, 364  (A, C),  398, 396  (B, D) | 354, 365  (A, C),  398, 398  (B, D) |
| R.m.s.d., bond lengths (Å) | 0.008 | | 0.008 | | 0.008 | 0.008 | 0.008 | 0.008 |
| R.m.s.d., angles (°) | 0.92 | | 0.92 | | 0.96 | 0.93 | 0.98 | 0.97 |
| Ramachandran favored (%) | 96.4 | | 96.4 | | 96.6 | 97.9 | 95.8 | 96.5 |
| Ramachandran allowed (%) | 3.0 | | 3.4 | | 3.1 | 2.0 | 4.2 | 3.6 |
| Ramachandran outliers (%) | 0.6 | | 0.2 | | 0.3 | 0.1 | 0 | 0.1 |
|  |  |  | |  |  |  |  |  |
| Average *B* factor (Å^2^) |  |  |  |  |  |  |  |  |
| Overall | 35.0 | | 30.3 | | 33.4 | 61.7 | 85.1 | 72.0 |
| Macromolecules | *33.6* | | *29.3* | | *33.3* | *61.7* | 85.6 | 72.8 |
| Ligands | *24.3* | | *23.5* | | *25.2* | *51.6* | 77.1 | 56.0 |
| Solvent | *43.8* | | *40.0* | | *41.5* | *63.6* | 68.4 | 59.7 |
| No. of TLS groups | 2 | | 1 | | 3 | 4 | 4 | 4 |
| **PDB code** | **8BJH** | | **8BJI** | | **8BJJ** | **8BR1** | **8BO1** | **8BR0** |

(Values in parentheses are for the highest resolution shell).

^#^: First and last indicated residues in Vn-ExoY constructs are from the Uniprot A0A6N3LUE9_9VIBR sequence from *Vibrio nigripulchritudo* MARTX toxin.
