# Supplementary figures and images for: Functional and structural insights into the multi-step activation and catalytic mechanism of bacterial ExoY nucleotidyl cyclase toxins bound to actin-profilin
Source: PLoS Pathog. 2023 Sep 25;19(9):e1011654. doi: 10.1371/journal.ppat.1011654 (PMC10553838; doi:10.1371/journal.ppat.1011654)

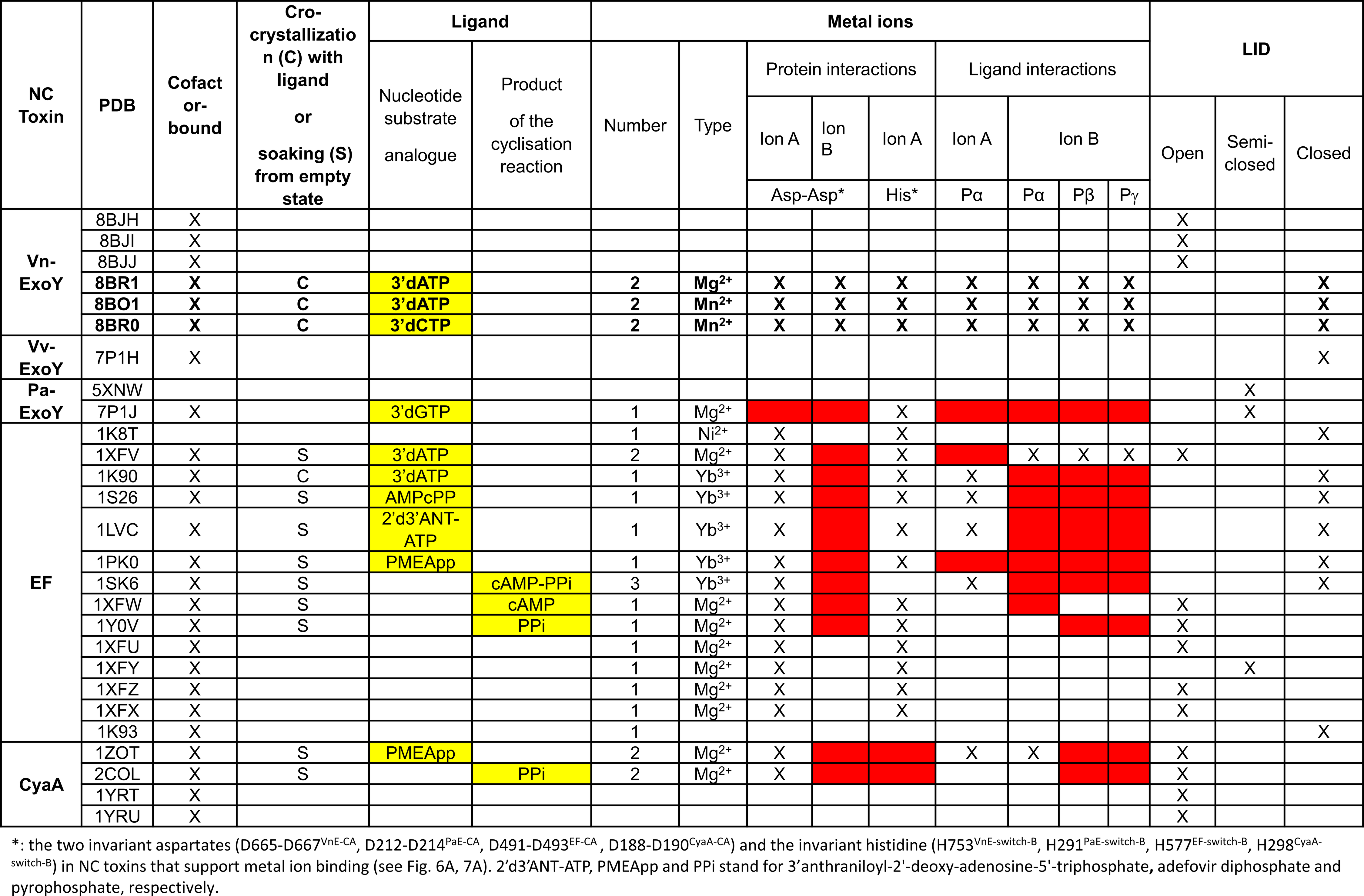

Supplement: S3 Table — Structures of cofactor-activated NC toxins containing a nucleotide substrate analogue or products of the cyclisation reaction are highlighted in yellow. These structures for Pa-ExoY, EF and CyaA have been compared with the activated Vn-ExoY structures bound to 3’dATP or 3’dCTP. Where one of the mechanistic features of the NBP of Vn-ExoY is not conserved in other NC toxin structures bound to a substrate analogue or to products of the cyclisation reaction, the box in the table corresponding to that feature is coloured red to highlight the differences. No published structures of cofactor-bound NC toxins with a nucleotide substrate analogue of ATP/GTP bound in their NBP exhibit coordination and conformations mechanistically similar or equivalent to the crystal structures of actin-activated Vn-ExoY utilizing 3’dATP or 3’dCTP as bound nucleotide substrate analogues with two metal ions (presented here). (TIF) [file ppat.1011654.s003.tif]

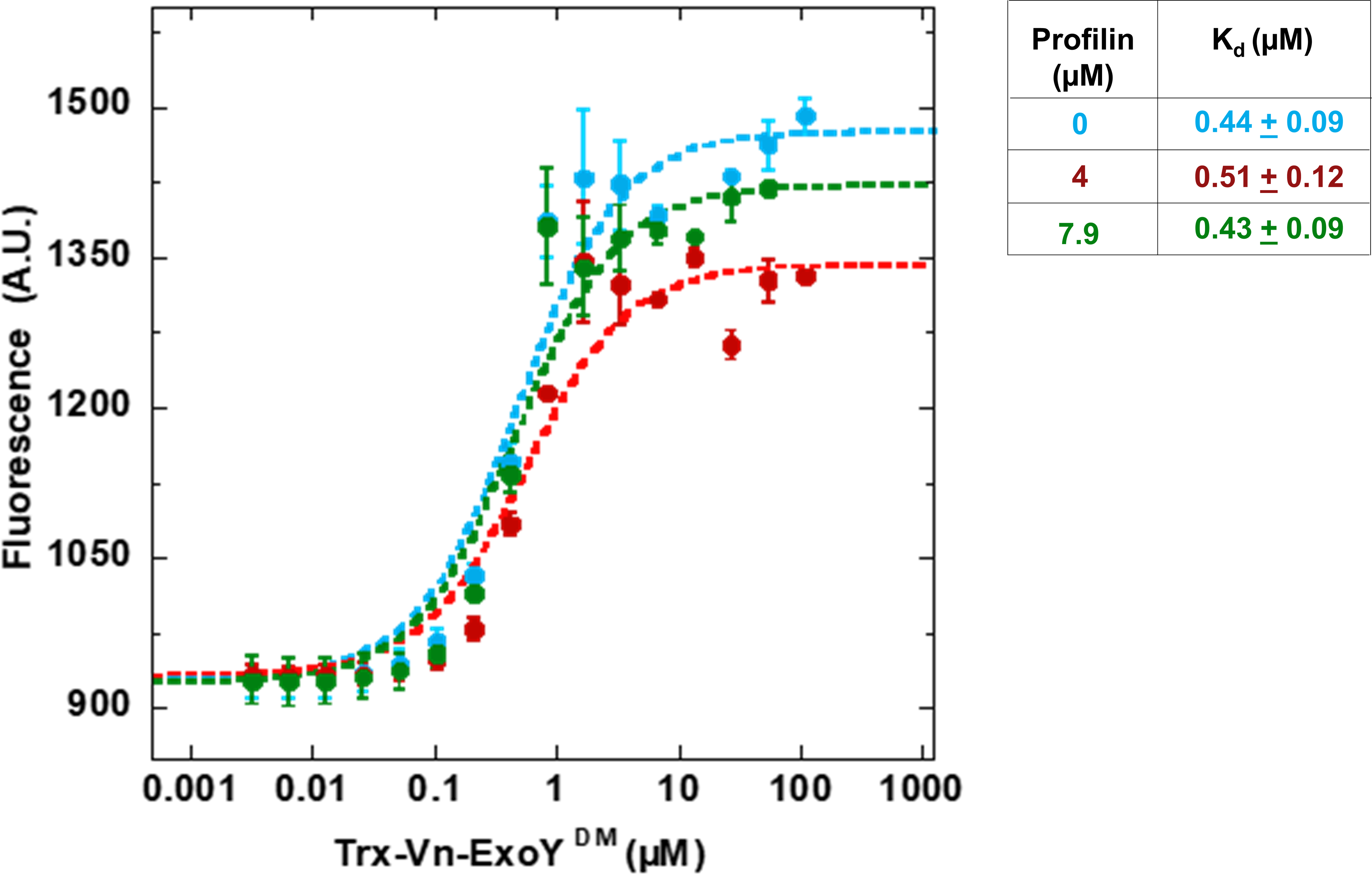

Supplement: S1 Fig — Titration of free or profilin-bound Alexa-488-labelled ATP-G-actin with unlabelled Trx-Vn-ExoYDM and determination of Kd values from changes in microscale thermophoresis (MST) intensities. 0.075 μM Alexa-488-labelled, latrunculin-A-bound G-actin alone/free (cyan) or bound to 4 (red) and 7.9 (green) μM profilin was titrated with Trx-Vn-ExoYDM concentrations as indicated. This corresponds to a 2-fold dilution series from 108 to 0.033 μM with raw data in an insert. Error bars are s.d. (n≥3). The experiment using either the changes in MST (here) or fluorescence (Fig 2C) intensity gave very similar Kd. The Kds averaged over both experiments (MST and fluorescence) were for Trx-Vn-ExoYDM binding to: free G-actin (cyan): Kd = 0.44 ± 0.09 μM), G-actin bound to 4 μM profilin (red): Kd = 0.51 ± 0.12 μM, and G-actin bound to 7.9 μM profilin (green): Kd = 0.43 ± 0.09 μM. (TIF) [file ppat.1011654.s006.tif]

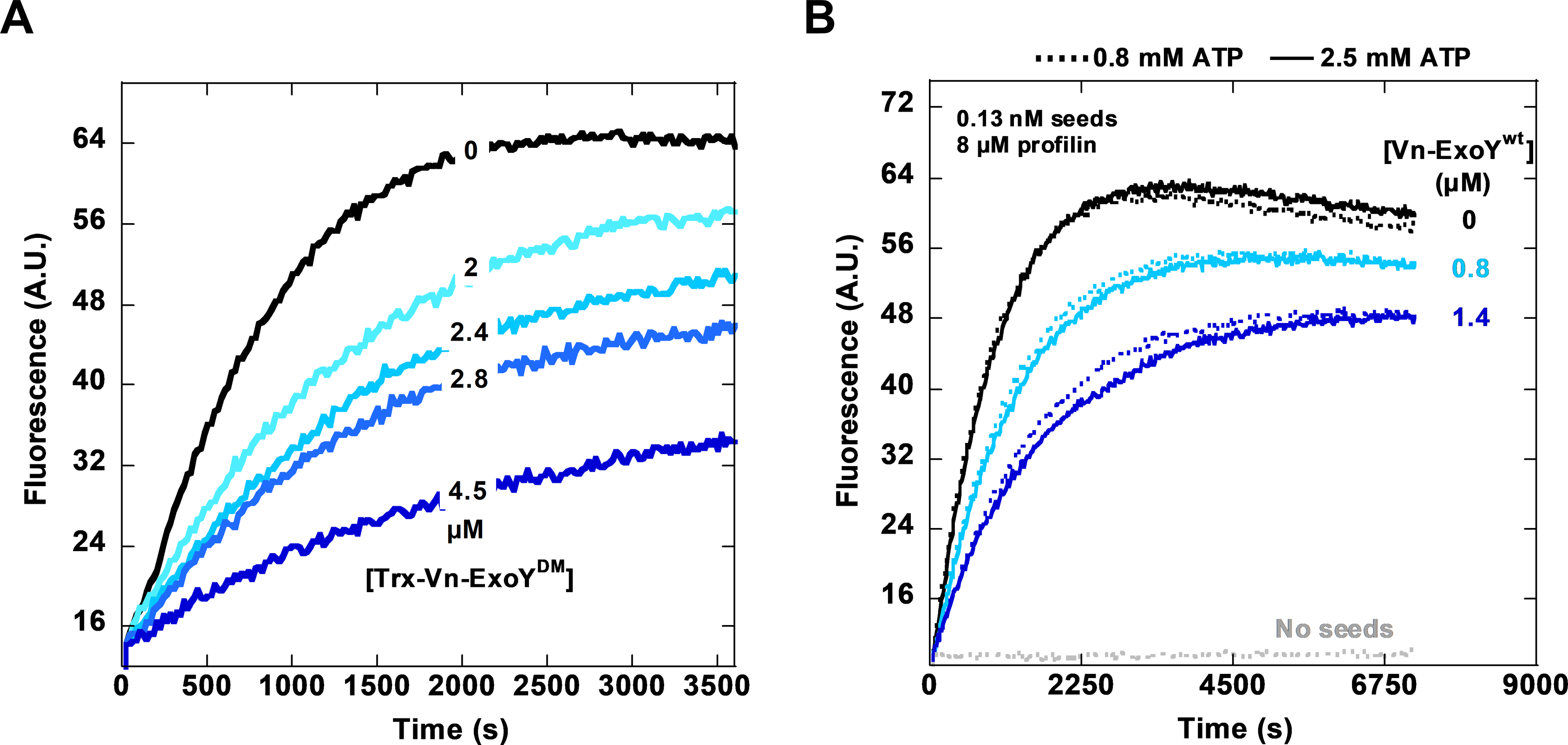

Supplement: S2 Fig — A) Barbed end growth from spectrin-actin seeds (0.22 nM) was measured using 1 μM G-actin (5% pyrenyl-labelled), 8 μM profilin, and the indicated concentrations (μM) of the inactive Trx-Vn-ExoYDM construct. Trx-Vn-ExoYDM inhibits barbed-end elongation from profilin-actin like Vn-ExoYwt, demonstrating that Vn-ExoY-mediated effects on actin polymerisation are independent of its AC activity. The buffer was 50 mM KCl, 2 mM MgCl2, 1 mM ATP, 15 mM Tris-HCl pH 7.8, 0.5 mM CaCl2, and 1 mM TCEP. B) Barbed end growth from spectrin-actin seeds (0.13 nM) was measured using 1 μM G-actin (5% pyrenyl-labelled), 8 μM profilin, and the indicated concentrations (μM) of active Vn-ExoYwt with either 0.8 (dashed lines) or 2.5 (solid lines) mM ATP. No differences in actin polymerisation kinetics were seen at different ATP concentrations. This indicates that the inhibitory effects of Vn-ExoY on actin polymerisation kinetics are independent of its AC activity throughout the duration of the experiments. Single experiments of actin polymerisation kinetics are shown. They are representative of a minimum of 3 independent experiments. (TIF) [file ppat.1011654.s007.tif]

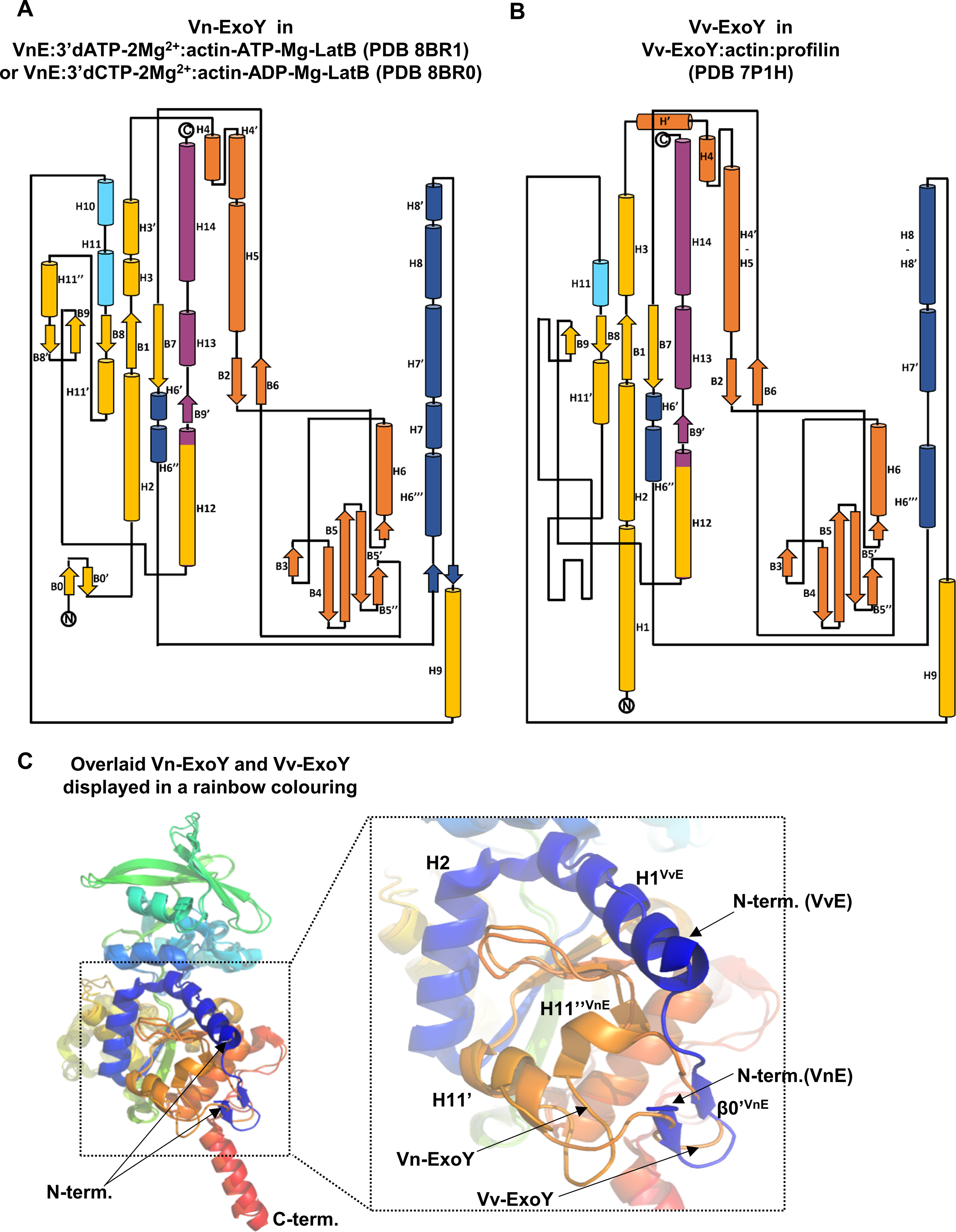

Supplement: S3 Fig — Topology diagrams of (A) Vn-ExoY in PDB: 8BR1, 8BO1 or 8BR0, and (B) Vv-ExoY in PDB 7P1H [28]. (A) The structural topologies correspond in (A) to Vn-ExoY bound to actin-ATP-LatB and 3’dATP-2Mg2+ (PDB 8BR1, 2.0 Å resolution), actin-ATP-LatB and 3’dATP-2(Mn/Mg)2+ (PDB 8BO1, 2.5 Å resolution) or actin-ADP-LatB and 3’dCTP-2Mn2+ (PDB 8BR0, 2.2 Å resolution), and in (B) to Vv-ExoY bound to actin-ATP-LatB and profilin (PDB 7P1H). In this 3.9-Å resolution cryo-EM structure, the 3’dATP ligand used for the preparation of the complex could not be modelled in Vv-ExoY. The amino-acid sequences of the Vn- and Vv-ExoY homologues are 89% similar. However, their structures show a difference in the structural topology of the main chain trace at two segments of their sequence. (C) The superimposed structures of Vn-ExoY and Vv-ExoY, shown in a rainbow of colours (from blue to red from N- to C-terminus, respectively), with their N-terminus enlarged in the right inset. The backbone trace of Vn-ExoY N-terminal sequence Y468QSRDLVLEP477 overlaps with a small region located further in Vv-ExoY backbone. This overlapping region is located between switch B and C (namely between helix H11’ and β-stand B9) and corresponds to the Vv-ExoY sequence L332GEGKGSIQT341. As a result, the backbone trace of their N-termini (Vn-ExoY sequence K466TYQSRDLVLEPIQHPKSIEL486, Vv-ExoY sequence S19RDLVLEPIVQPETIEL34) is very different, as is the backbone trace of their region between helix H11’ and β-stand B9 (Vn-ExoY sequence D781DGLGEGKGSIQT793, Vv-ExoY sequence E329DGLGEGKGSIQT341). The ab initio protein structure prediction of Vv-ExoY using the AlphaFold protein-prediction tool [68] suggests that Vv-ExoY adopts a backbone trace topology similar to that observed for Vn-ExoY in the crystal structures. Vv-ExoY bound to actin-ATP:profilin [28] and Vn-ExoY bound to actin-ATP/-ADP and 3’dATP/3’dCTP have otherwise similar overall conformations, including at their switch A, with a r.m.s.d. of 1.4 Å for 364 overlaid Cα [file ppat.1011654.s008.tif]

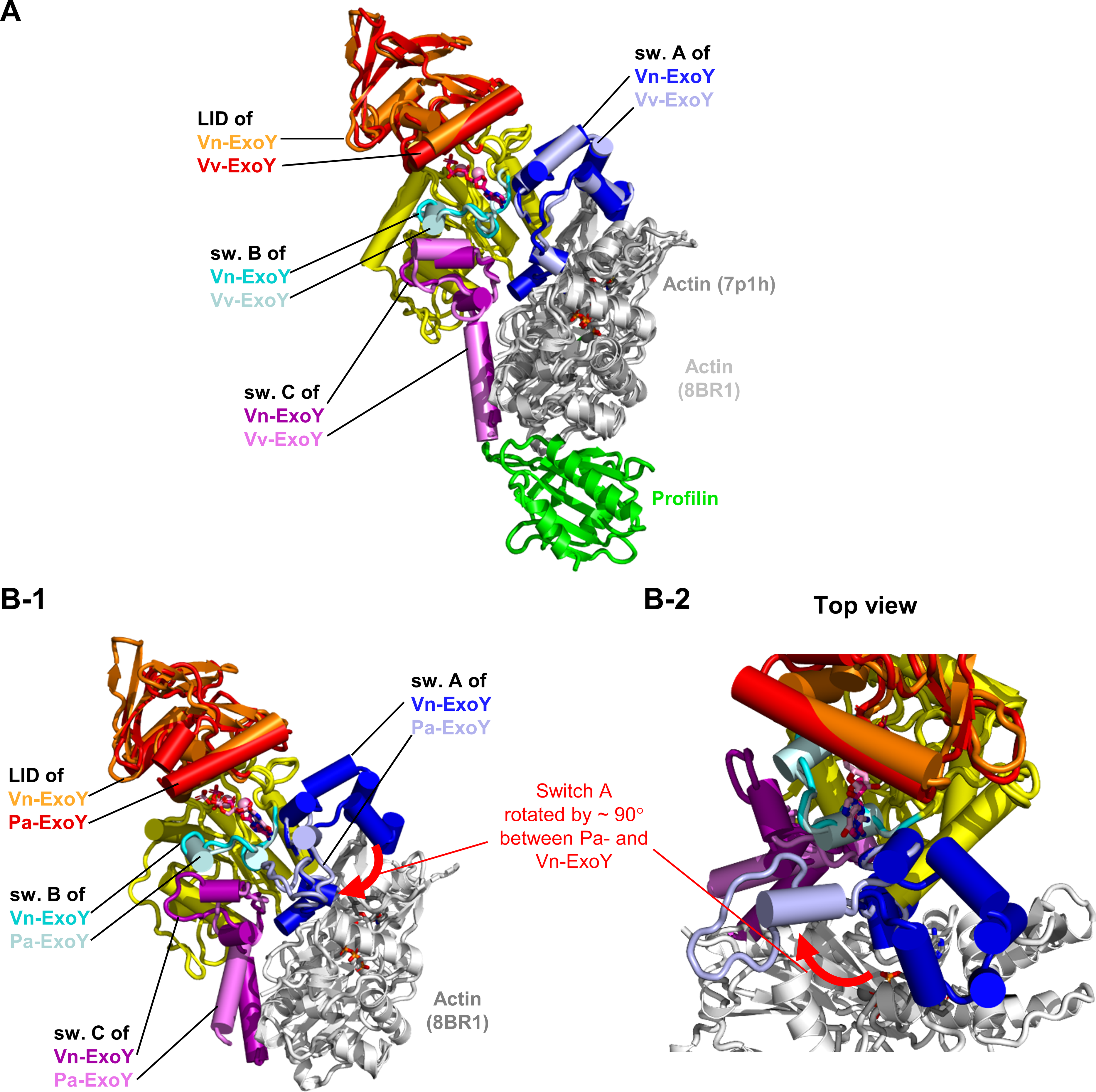

Supplement: S4 Fig — Overlays of the Vn-ExoY:3’dATP-2Mg2+:actin-ATP-LatB structure (2.1 Å resolution crystal structure, PDB: 8BR1) with (A) the Vv-ExoY:actin-ATP:profilin structure (3.9 Å resolution cryoEM structure, PDB: 7P1H) [28] and (B) Pa-ExoY structure from the Pa-ExoY:3’dGTP-1Mg2+:F-actin-ADP-Pi complex (3.2 Å resolution cryoEM structure, PDB: 7P1G) [28]. The structures are superimposed on their CA subdomain (residues 468-532Vn-ExoY-CA and 663-849Vn-ExoY-CA). Panels (A) and (B-1) show the same side views of the complexes, while (B-2) is a top view of (B-1). (TIF) [file ppat.1011654.s009.tif]

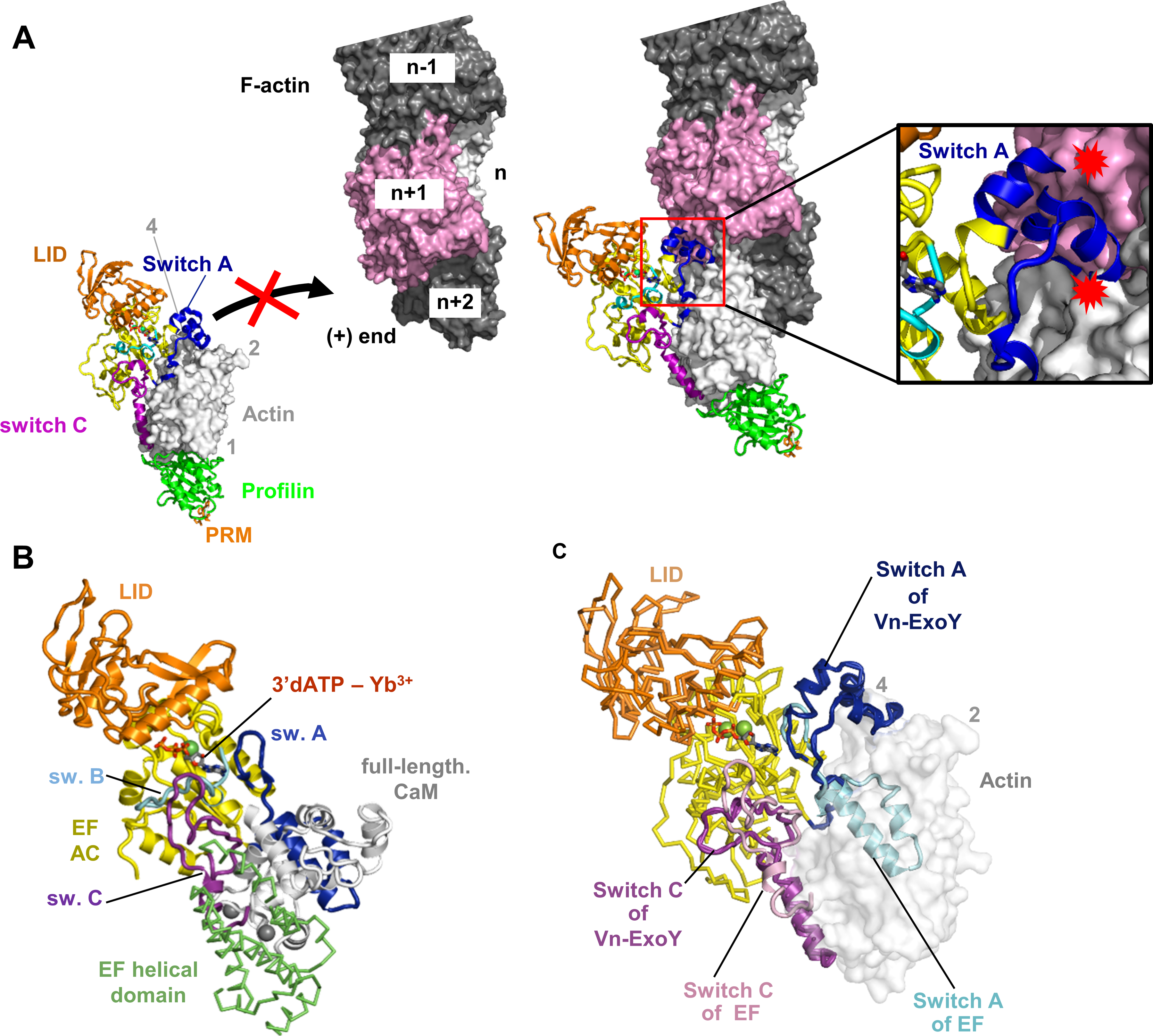

Supplement: S5 Fig — A) Docking of the Vn-ExoY-3’dATP-2Mg2+:actin-ATP structure in complex with profilin and PRM to F-actin (PDB code: 6FHL) shows that the interaction of the Vn-ExoY switch A between actin subdomains 2 and 4 prevents bound actin (see Fig 4C) from assembling at the barbed (+) end of F-actin. It induces important steric clashes of switch A (red explosion symbols) with the penultimate actin subunit (in pink) at the barbed-end. Switch A binding to actin is also the region most incompatible with longitudinal contacts between adjacent actin subunits of the same F-actin strand (white and pink actin). This also most inhibits Vn-ExoY binding along F-actin [5,28]. B) Cartoon representation of the 3’dATP-EF-CaM complex. The NC catalytic domains of EF, CyaA and Vn-ExoY use the same regions, i.e. switch A and C, and a similar orientation to interact with their cofactor. The C-terminal Ca2+-binding globular domain of the 8.4-kDa protein CaM is responsible for most of the interactions with the EF or CyaA switch A and C [24,25,27]. However, it is much smaller than 42-kDa actin. It therefore only overlaps with actin subdomains 1 and 3. C) Structural comparison of G-actin- and CaM-activated Vn-ExoY and EF conformations, respectively. The common catalytic core, CA (yellow) and CB (orange) domains are shown in a cartoon tube representation, and the regions determinant for cofactor specificity, switch A (blue and cyan) and C (pink and purple) regions, are shown in a cartoon representation. The positioning of switch A in EF or CyaA bound to CaM and in Vn-ExoY or Pa-ExoY bound to G or F-actin, respectively, is the most divergent region at the cofactor-toxin binding interface. (TIF) [file ppat.1011654.s010.tif]

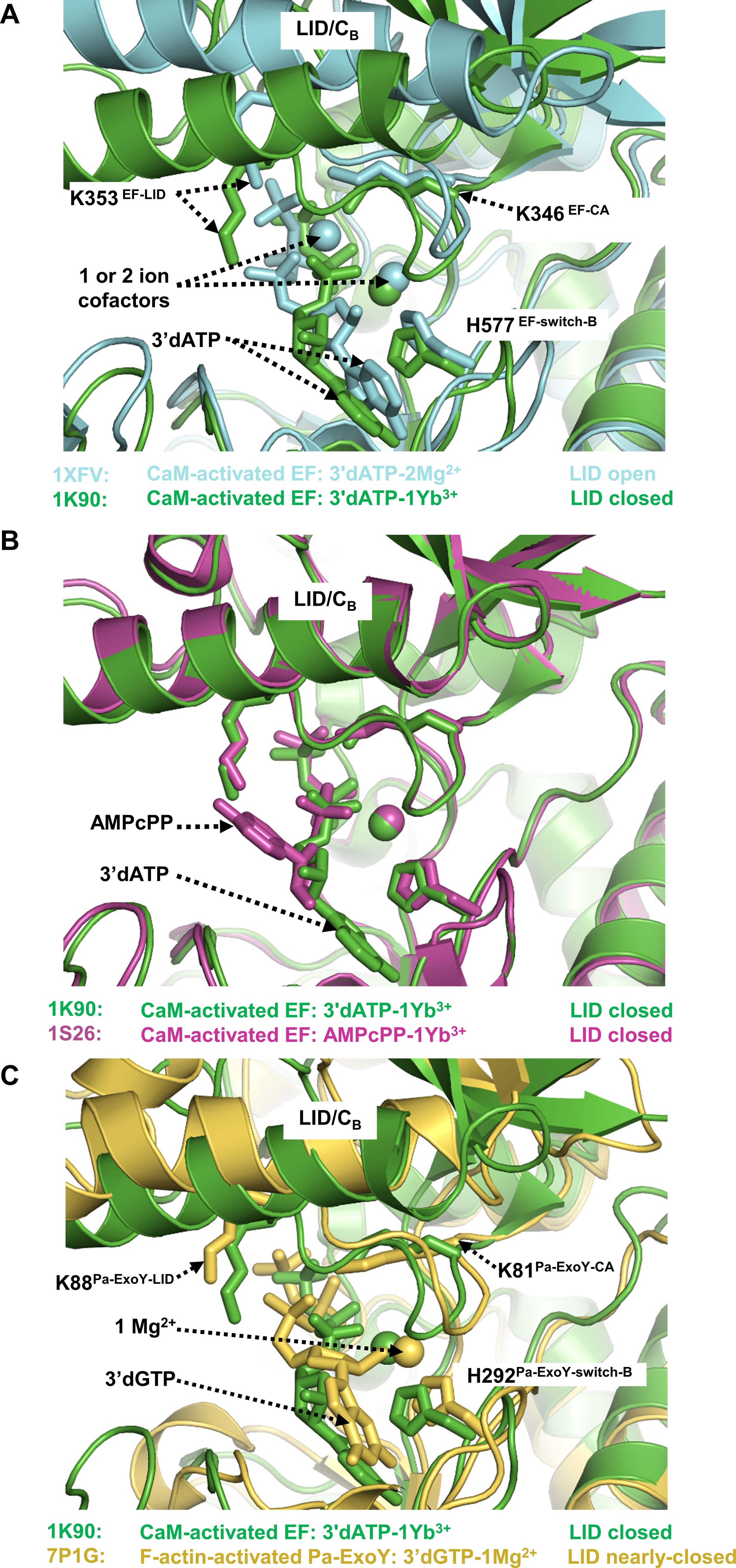

Supplement: S6 Fig — The structures of EF bound to CaM and ATP analogue (A, B) or Pa-ExoY bound to F-actin and GTP analogue (C) show strong active-site structural heterogeneity. They show either 1 or 2 metal ions associated with the substrate analogue, variable conformation or positioning of the different moieties of the purine nucleotide substrate analogues, and different positions of the LID/CB subdomain relative to the CA subdomain. The protein structures are shown in cartoon and coloured in cyan (PDB: 1XFV [27]), green (PDB: 1K90 [24]), light magenta (PDB: 1S26 [29]) and yellow (PDB: 7P1G [28]). The catalytic domains of the EF and Pa-ExoY NC toxins are superimposed on their CA subdomain. Metal ions are shown as spheres and the ligands and side chains of important catalytic residues (K346EF-CA/K81Pa-ExoY-CA, H353EF-LID/K88Pa-ExoY-LID and H577EF-Switch-B/H292Pa-ExoY-Switch-B) are shown as sticks. (A) Overlay of the CaM-activated structures of EF bound to 3’dATP containing 1 or 2 metal cofactors. (B) Overlay of the CaM-activated structures of EF bound to ATP analogues containing 1 metal cofactor. (C) Overlay of EF CaM-activated structure bound to 3’dATP with 1 Yb3+ metal ion from (A and B) and Pa-ExoY F-actin-activated structure bound to 3’dGTP with 1 Mg2+ metal ion. S7 Fig complements S6 Fig, offering together unambiguous evidence of the variations in the number of ions, the position and coordination of metal ion(s), and the position, conformation, and coordination of nucleotide substrate analogues. This includes variations in the coordination between the enzyme and nucleotide, as well as between the nucleotide and the metal ion(s). For instance, panel S6-B illustrates that the adenine ring of the AMPCPP bound to CaM-bound EF (PDB 1S26) is rotated approximately 180° compared to 3’dATP bound to CaM-bound EF (PDB 1XFV, 1K90) concerning the nucleotide conformation. Moreover, panels S6-A and S6-C demonstrate that 3’dATP (S6A Fig) or 3’dGTP (S6B Fig) can be bound with either 1 or 2 metal i [file ppat.1011654.s011.tif]

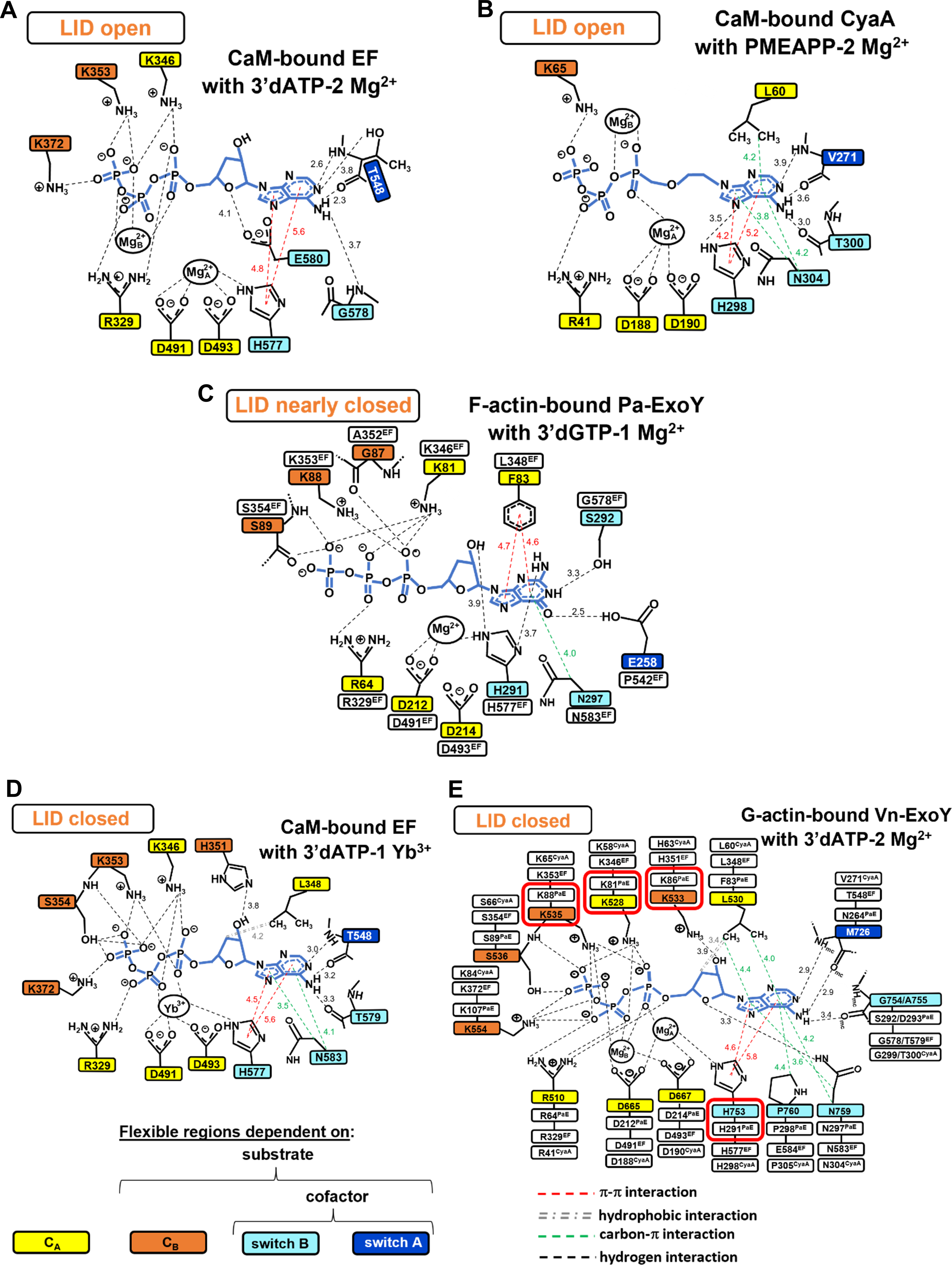

Supplement: S7 Fig — The active site of (A) CaM-activated EF with 3′dATP and two Mg2+ metal ions (3.35 Å resolution crystal structure, PDB: 1XFV) [25], (B) CaM-activated CyaA structure with adefovir diphosphate (9-(2-(phosphonomethoxy)ethyl)adenine diphosphate, or PMEAPP) and two Mg2+ metal ions (2.20 Å resolution crystal structure, PDB: 1ZOT) [27], (C) F-actin-activated Pa-ExoY with 3′dGTP and a single Mg2+ metal ion (cryoEM structure at an average 3.20 Å resolution, PDB: 7P1G) [28], (D) CaM-activated EF structure with 3′dATP and a single Yb3+ metal ion (2.75 Å resolution crystal structure, PDB: 1K90) [24], and (E) G-actin-activated Vn-ExoY with 3′dATP and two Mg2+ metal ions (2.04 Å resolution crystal structure presented in this article, PDB: 8BR1). Thresholds for interaction detection are those of the PLIP (protein-ligand interaction profiler) [88] and Arpeggio [89] web servers, and those from the PoseView [99] tool available on the ProteinsPlus web server [90,100]. S8 Fig shows the position of the LID/CB relative to the CA subdomain in PDBs 1XFV (A), 1ZOT (B) and 1K90 (D). The use of an unconventional metal ion such as Yb3+ for CaM-bound EF crystallisation is expected to alter the coordination of EF NBP with ATP only slightly compared to the putative physiological metal-ligand Mg2+, as the metal Yb3+ only slightly reduces the AC catalytic activity of EF [24]. (TIF) [file ppat.1011654.s012.tif]

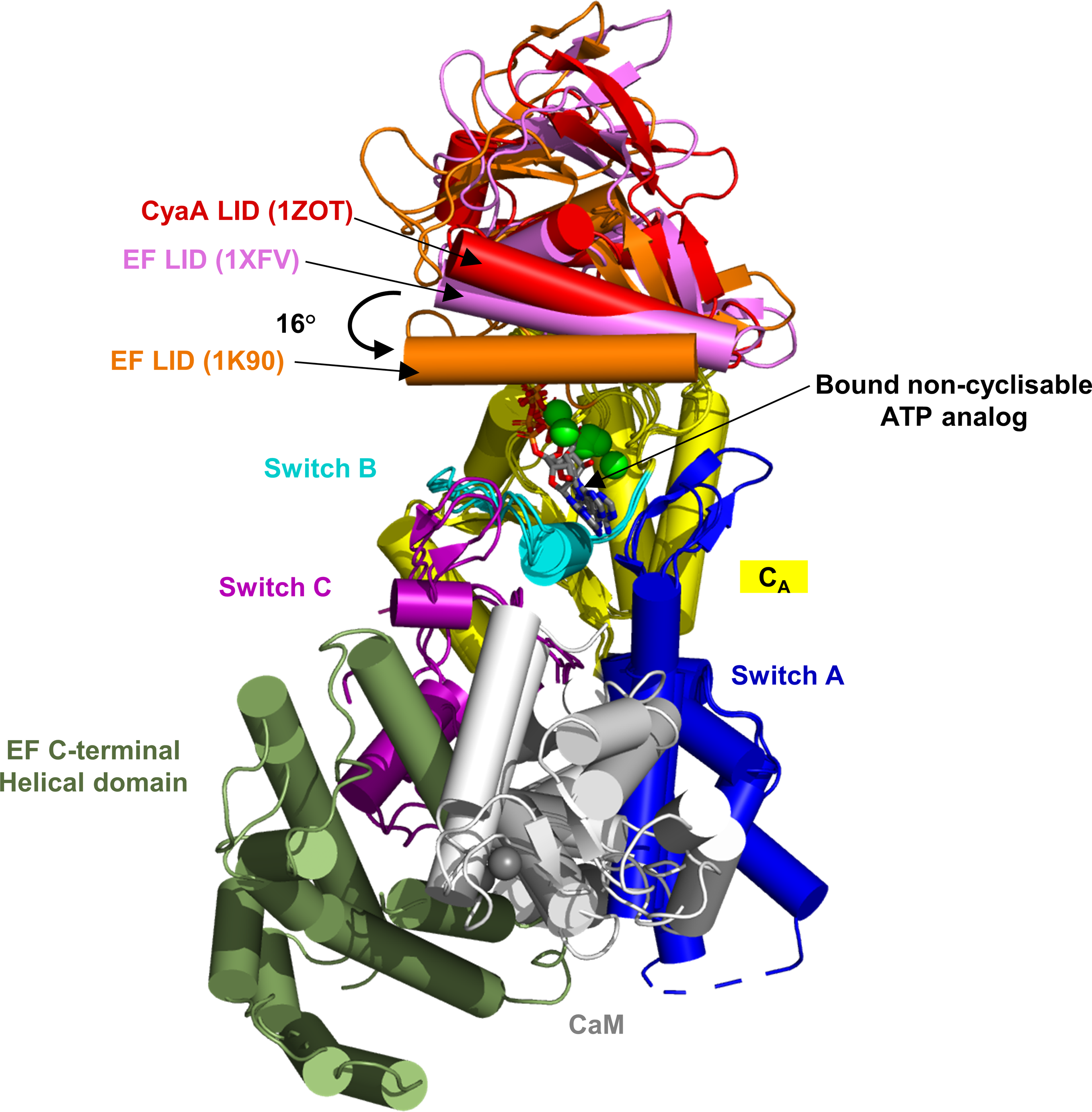

Supplement: S8 Fig — The EF structure with the LID in a closed conformation (1K90) and the EF and CyaA structures with the LID in an open conformation (1XFV and 1ZOT, respectively) are superimposed on their CA subdomains (in yellow). Protein domain motion analysis between the two structures of EF bound to CaM and 3’dATP (PDB 1K90 and 1XFV) shows that the LID/CB subdomain undergoes a 16° rotation as a rigid body around hinge regions 350-351EF and 488-489EF (motion analysis performed with DYNDOM [86]). Switch A, B, and C regions of EF and CyaA are coloured in blue, cyan, and purple, respectively. The LID region is shown in orange (1K90), pink (1XFV), and red (1ZOT). CaM and the additional C-terminal helical domain of EF are shown in white and green, respectively. Switch A exhibits minimal stabilisation (or only pre-stabilisation) by actin in the absence of substrate (Figs 3D–3F and 4D). On the other hand, EF and CyaA present a fully folded switch A in their nucleotide-free, CaM-bound state [24,25]. However, their CB/LID subdomain displays flexibility and can adopt various positions in isolated CyaA [53] or in different crystal structures of CaM-bound EF (as shown here and in Fig 6E). Numerous crystal structures of CaM-activated EF or CyaA, with nucleotide ligands bound into their active site, were crystallised without substrate analogues or reaction products and subsequently exposed to these ligands. The crystal packing in these structures may have hindered the complete rearrangement of the NBP and LID/CB subdomain closure (S3 Table). Consequently, due to the inherent flexibility of the LID/CB subdomain, both substrate analogues and metal ions occupy significantly distinct positions in the NBP of CaM-bound EF or CyaA (S6–S8 Figs) [1]. These distinct structures likely correspond to intermediate stages of LID/CB movement and substrate entry. (TIF) [file ppat.1011654.s013.tif]

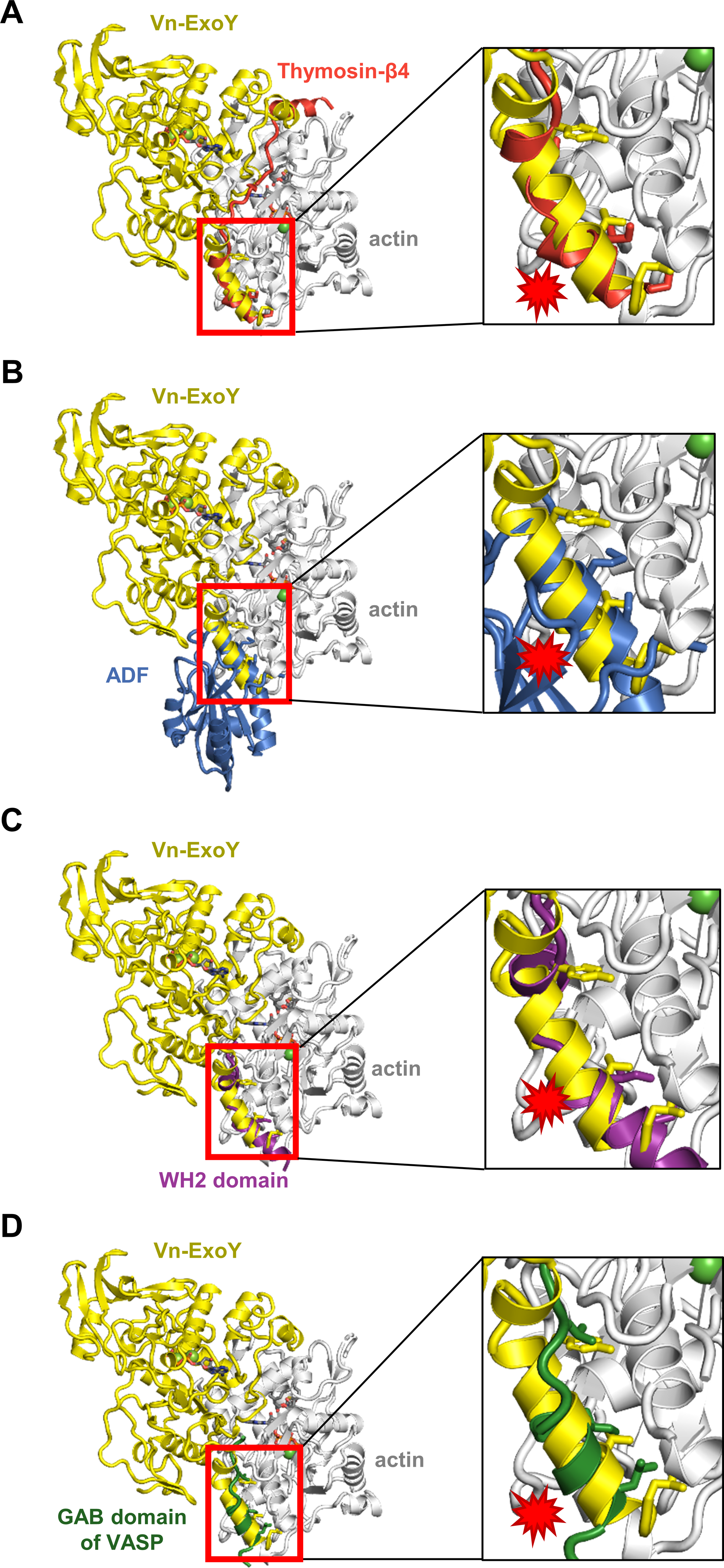

Supplement: S9 Fig — Binding interface overlap between the binding of Vn-ExoY switch C and the binding of major G-actin-binding (GAB) proteins/domains: (A) Thymosin-β4 (Tβ4) (PDB: 4PL7 [101]), (B) ADF (PDB: 3DAW [102]), (C) Cordon-bleu WH2 domain (PDB: 5YPU [103]), (D) GAB domain of VASP (PDB: 2PBD [79]). The interaction of the C-terminal amphipathic α-helix of Vn-ExoY switch-C within the hydrophobic cleft between actin subdomains 1 and 3 (shown in the right zoom panel) is a common binding site of actin-binding proteins (ABPs) [104]. Apart from profilin, this interaction of the Vn-ExoY switch-C C-terminus competes with a similar interaction that exists with most G-ABPs as shown in these examples. The structural models are consistent with Fig 2A in the main text, which shows the competition in solution between Vn-ExoY and the Tβ4 or Cordon-bleu WH2 domain for binding to G-actin. Taken together, these data suggest that the G-actin:profilin complex serves as a physiological cofactor for Vn-ExoY and its closely-related ExoYs (Figs 5D and S10) in eukaryotic cells. (TIF) [file ppat.1011654.s014.tif]

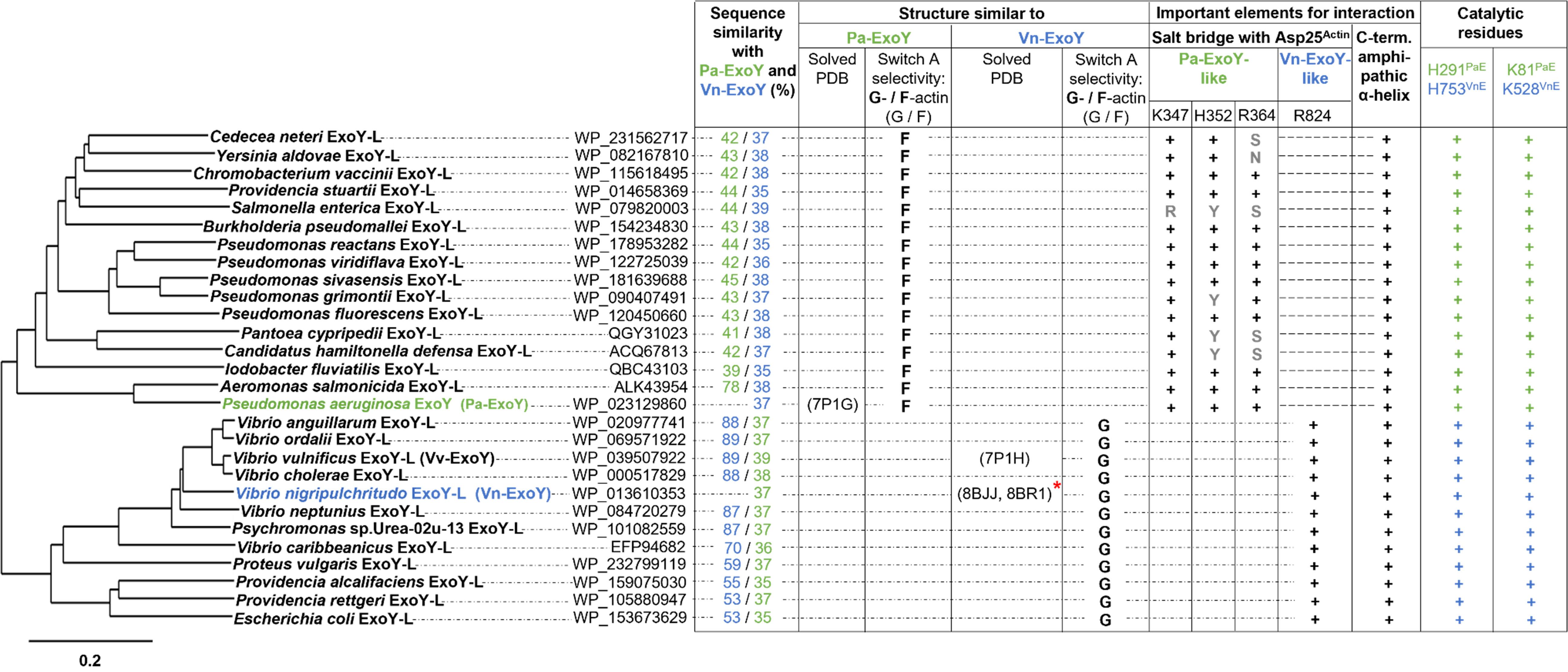

Supplement: S10 Fig — ExoYs are classified as Vn-ExoY-like or Pa-ExoY-like homologues based on both their sequence similarity and predicted structure. Except for the indicated PDBs, the ab initio protein structure of all other ExoYs was predicted using the highly-accurate protein prediction tool AlphaFold [68]. The amino-acid sequence alignment of Pa-ExoY, Vn-ExoY and ExoY-like proteins/modules found in other γ- or β-proteobacteria and potentially activated by actin was performed using Clustal Omega (http://www.ebi.ac.uk, accessed on 12 September 2022) [105]. The multiple sequence alignment from Clustal Omega was used to construct the phylogenetic tree by using TreeDyn (www.phylogeny.fr) [106]. Pairwise sequence similarities (%) with Pa-ExoY and Vn-ExoY are shown in green and blue, respectively (Sequence Identity And Similarity (SIAS) tool, http://imed.med.ucm.es/Tools/sias.html accessed 12 September 2022). The NCBI accession numbers of the protein sequences are given to the right of the phylogenetic tree. The structures of Vn-ExoY solved in this study with actin or actin:profilin are marked with a red asterisk. The selectivity of the switch A conformation for G- or F-actin in the solved or AlphaFold-predicted structures of the ExoYs is indicated by G or F, respectively. The predicted structures of the ExoYs, which are closely-related to the MARTX Vn-ExoY module, are compatible with the formation of an ExoY:actin:profilin ternary complex and a conformation of switch A on actin that inhibits G-actin:profilin assembly at the barbed-ends of actin filaments. A + sign indicates that the Vn-ExoY or Pa-ExoY residue/structural element is conserved at the same position in the ExoY-like homologue’s sequence and predicted structure. (TIF) [file ppat.1011654.s015.tif]

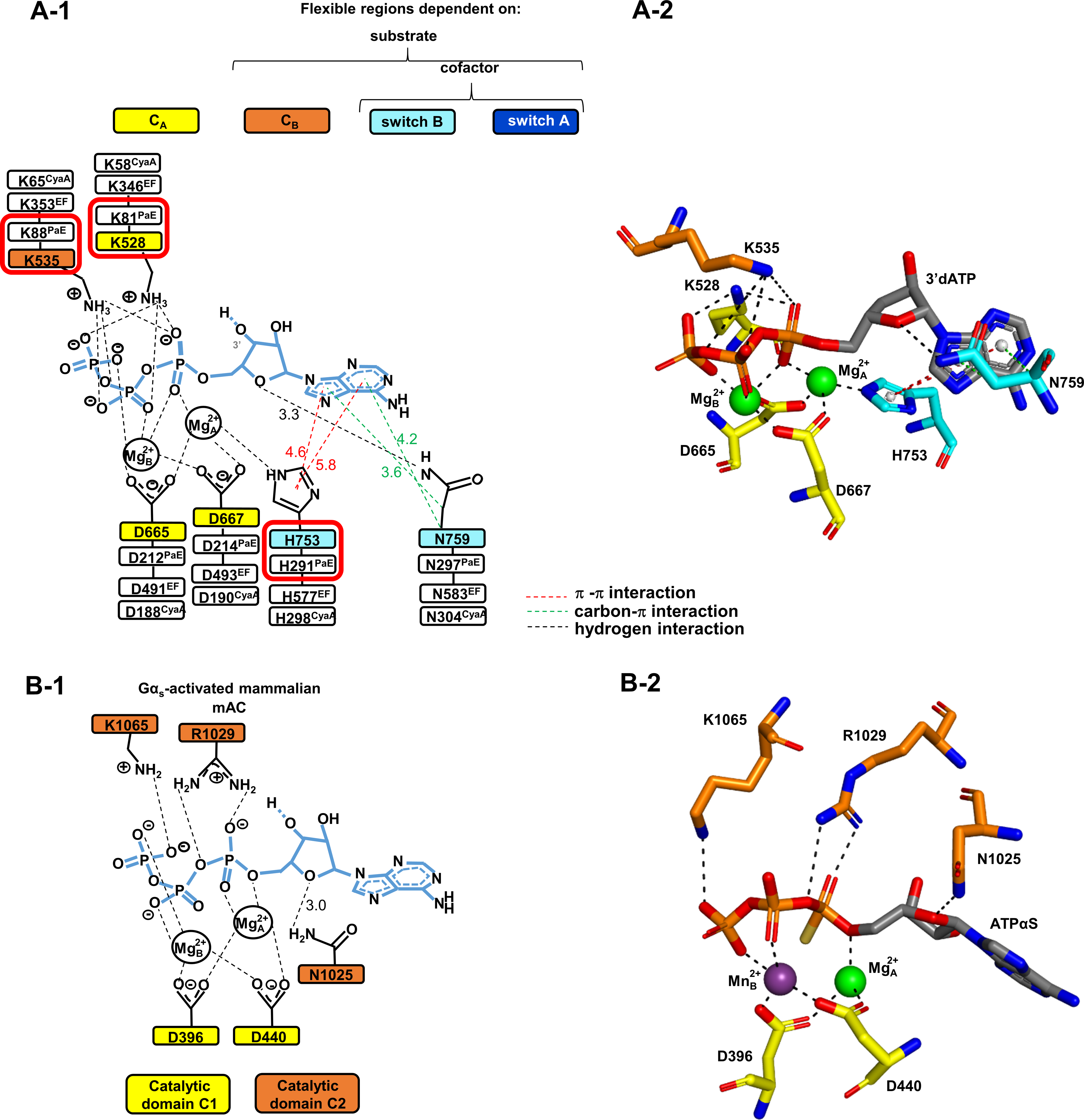

Supplement: S11 Fig — Protein-ligand interactions of the ATP analogue and metal ions in the active site of Vn-ExoY class II AC (A, PDB: 1BR1) and mammalian transmembrane class III AC (tmAC) (B, PDB: 1CJK) activated by their respective cofactors are shown. (A-1) A simplified model of ATP binding in activated Vn-ExoY is presented, based on its interactions with 3’dATP in the Vn-ExoY-3’dATP-2*Mg2+:ATP-actin-LatB structure. (A-2) Three-dimensional protein-ligand interactions in the corresponding crystal structure. (B-1) A simplified model of ATP binding in activated class III tmAC is presented, based on the structure of the type V AC C1a/type II AC C2 heterodimer bound to the ATP analogue RP-ATPαS (PDB 1CJK). (B-2) Three-dimensional protein-ligand interactions in the corresponding crystal structure. For clarity and simplicity, only a few residues crucial for the purinylyl cyclase activities of Vn-ExoY and tmAC are shown here, while the full protein-ligand interactions are illustrated in Fig 6A and 6F. The thresholds used for interaction detection follow the criteria of the PLIP [11] and Arpeggio [12] web servers, along with the PoseView tool in the ProteinsPlus web server [14,15]. Regarding the catalysis mechanism of tmAC, a computational study [42] has proposed a substrate-assisted general base catalysis, wherein several residues of the enzyme with metal-ion cofactors play essential roles in reducing the two highest energy barriers of the reaction. Specifically, K1065tmAC-IIC2, Mg2+A, Mg2+B and the two conserved D96D440tmAC-VC1 anchoring metal ions are critical in facilitating the proton transfer from the ribosyl 3′O to a γ-phosphate oxygen [42]. Subsequently, R1029tmAC-IIC2 is identified as critical for the concerted phosphoryl transfer step, with K1065tmAC-IIC2 also involved. Further investigation is required to determine the significance of H753VnE-switch-B (or its equivalent histidine in other NC toxins) in initiating 3’OH deprotonation via Mg2+A and whether K528VnE-CA (or equivalent) i [file ppat.1011654.s016.tif]

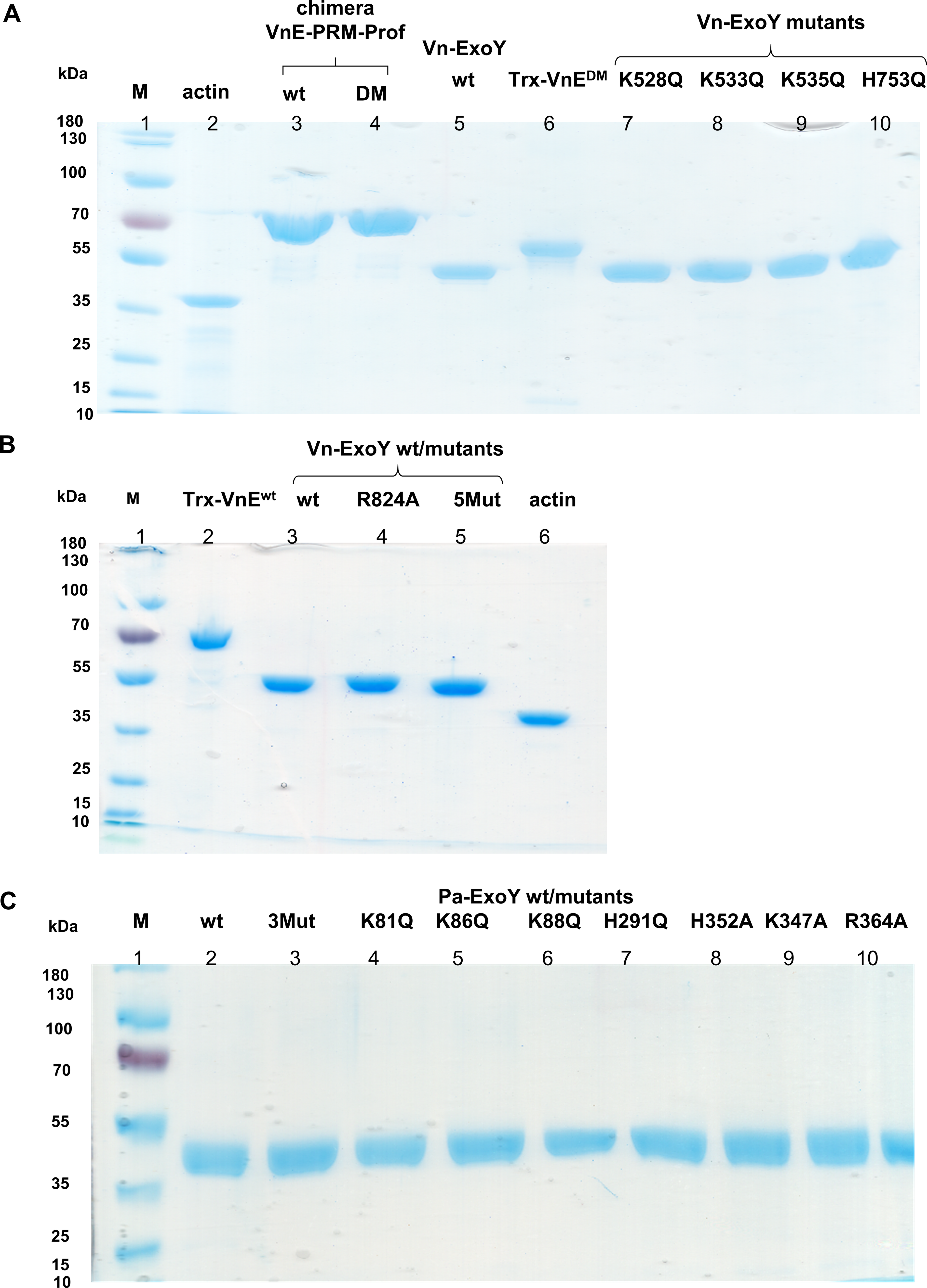

Supplement: S12 Fig — Molecular mass markers (in kDa) are shown on the left A–C (M, lane 1). (A), lanes 2–10: purified actin, chimera Vn-ExoY (wt/DM)-PRM-Prof, Vn-ExoYwt (residues 455–863), Trx-Vn-ExoYDM, Vn-ExoY mutants (residues 455–863): K528QVnE-CA, K533QVnE-LID, K535QVnE-LID, H753QVnE-switch-B, respectively. (B), lanes 2–6: Trx-Vn-ExoYwt, Vn-ExoYwt (residues 455–863), Vn-ExoY mutants (residues 455–863): R824AVnE-switch-C, 5 Mut (Y850A-R854A-V857A-K860A-L861A)VnE-switch-C and actin, respectively. (C), lanes 2–10: Pa-ExoY wt/Mutants (residues 20 to 378): Pa-ExoYwt, 3 Mut (F367A-L371A-F374A)PaE-switch-C, Pa-ExoY K81QPaE-CA, K86QPaE-Lid, K88QPaE-LID, H291QPaE-switch-B, H352APaE-switch-C, K347APaE-switch-C, R364APaE-switch-C, respectively. All samples were centrifuged at 16,000 g for 10 min before loading 3.5 μM, separating by 10% SDS-PAGE and staining with Coomassie blue. (TIF) [file ppat.1011654.s017.tif]

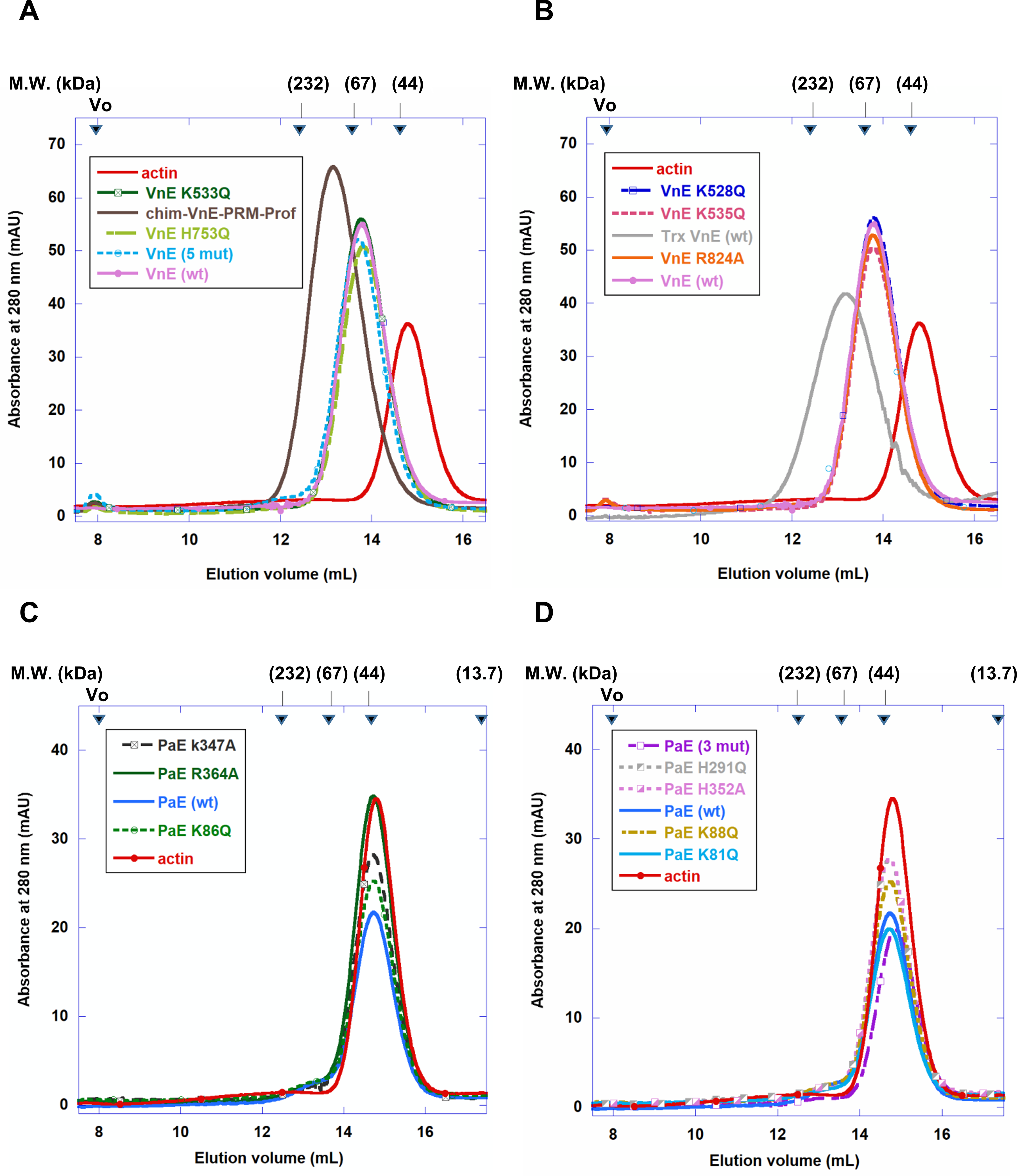

Supplement: S13 Fig — Elution profiles of (A-B) actin (42 kDa) and purified Vn-ExoYwt/mutant proteins (residues 455–863, 50 kDa), and (C-D) actin and purified Pa-ExoY wt/mutant proteins (residues 20 to 378, 44 kDa). The proteins are: (A) Latrunculin B-bound actin-ATP (elution volume of 14.79 mL), Vn-ExoY mutant K533QVnE-LID (13.76 mL), the chimera Vn-ExoYDM-PRM-Prof (72 kDa, 13.17 mL), Vn-ExoY mutants: H753QVnE-switch-B (13.80 mL), 5 Mut (Y850A-R854A-V857A-K860A-L861A) (13.70 mL) and Vn-ExoYwt (13.81 mL), (B) actin-ATP-LatB (14.79 mL), Vn-ExoY mutants K528QVnE-CA (13.79 mL), K535QVnE-LID (13.77 mL), Trx-Vn-ExoYDM (69 kDa, 13.20 mL), Vn-ExoY mutant R824VnE-switch-C (13.77 mL) and Vn-ExoYWT (13.81 mL), (C) Pa-ExoY mutants: K347APaE-switch-C, R364APaE-switch-C, Pa-ExoYwt, Pa-ExoY mutant K86QPaE-LID and actin-ATP-LatB (14.79 mL), (D) Pa-ExoY mutants: 3 Mut (F367A-L371A-F374A), H291QPaE-switch-B, H352APaE-switch-C, Pa-ExoYwt, Pa-ExoY mutants: Pa-ExoY K88QPaE-LID, K81QPaE-LID and actin-ATP-LatB (14.79 mL). All the Pa-ExoY wt/mutant proteins were eluted with a similar elution volume of 14.74 mL. The purified proteins were ultracentrifuged at 100,000 g for 20 min at 4°C and loaded as a volume of 200 μl at a concentration between 2 and 5 μM to distinguish between very similar elution profiles. Protein elution at 4°C was monitored by absorbance at 280 nm on a Superdex 200 HR 10/300 SEC column (~24 mL) equilibrated in (25 mM Tris-HCl pH 7.5, 150 mM KCl, 2 mM MgCl2 and 1 mM DTT). V0 is the void/dead volume of the column (7.9 mL). The elution volumes of the proteins used for column calibration are shown in brackets and indicated by black arrows at the top of each panel. These include catalase (Cat, 232 kDa, 12. 5 mL), bovine serum albumin (BSA, 67 kDa, 13.6 mL), ovalbumin (Ova, 44 kDa, 14.6 mL) and ribonuclease (RNase A, 13.7 kDa, 16.9 mL). (TIF) [file ppat.1011654.s018.tif]
