# Supplementary material for: Functional and structural insights into the multi-step activation and catalytic mechanism of bacterial ExoY nucleotidyl cyclase toxins bound to actin-profilin
Source: PLoS Pathog. 2023 Sep 25;19(9):e1011654. doi: 10.1371/journal.ppat.1011654 (PMC10553838; doi:10.1371/journal.ppat.1011654)
Supplement: Attachment — Submitted filename: PlosP-D2300717-SRS-review.docx [file ppat.1011654.s020.docx]

Teixeira-Nunas and colleagues present a comprehensive structural, biochemical and mutagenic analysis of the mechanism by which the class II nucleotidyl cyclase Vn-Exo-Y from to show how it is catalytically activated by interaction with G-actin or the G actin-profillin complex. The study is based on six medium (2.5Å) to high (1.6Å) resolution crystal structures, supported by assays of actin elongation, thermophoresis and equilibrium sedimentation to observe actin/profilin/Exo-Y assembly, and nucleotidyl cyclase assays of wild-type and mutant proteins. The authors report and discuss parallel experiments with the Pa-Exo-Y paralog, which interacts with F-actin rather than G actin are reported and discussed. The work is significant in that it contributes new and important insights to a growing body of literature that addresses that a large of family of class II AC bacterial toxins that weaken host cells by generating toxic concentrations of cyclic nucleotides, while interfering with other aspects of host cell regulation.

The major conclusions are:

(1) Vn-ExoY binds to actin monomers with ~20 uM affinity, but not to F-actin, and inhbits the assembly of actin at both the barbed (+) and pointed (-) ends of actin filaments. When fused to the Vn-ExoY complex profilin sequesters G actin with 100-fold higher affinity than Vn-ExoY alone.
(2) Binding of G-actin to Vn-ExoY enhances its catalytic nucleotidyl cyclase activity As in the case of G actin sequestration, profilin strongly enhances the apparent potency and efficacy of G actin activation of Vn-ExoY cyclase activity.
(3) Crystal structures demonstrate that binding of G actin to Vn-ExoY induce conformational changes in three switch regions and a rigid body rotation of a “lid” domain that enables binding of 3’-ATP and two Mg^2+^ cofactors, and productively orients essential catalytic residues .
(4) Despite considerable differences in the domain structure of transmembrane (and other) Class III adenylyl cyclases, the architecture of the Vn-ExoY active site (including Pa-ExoY and, other NC toxin cyclases for which structural data have been published) is similar to that of Class III cyclases and is likely to catalyze nucleotide cyclization by an analogous two-metal mechanism.
(5) The authors present the first structure of a class II NT cyclase bound to a 3’ pyrimidine nucleotide triphosphate analog, demonstrating a looser accommodation of the pyrimidine base than is observed for purine nucleotides.

Major comments

These conclusions are supported by the structural, mutagenic and actin-assembly and Cyclase assays. However, while error bars are shown for some of the assays (e.g., Figure 5) there is no discussion of the number of technical or “biological” replicates used in the various assays or methods used, if any, to validate the statistical significance of measured or derived rate constants presented in the manuscript.

The series of structures presented appear to demonstrate only two distinct conformational states, exemplified by VnE-PRM-Prof:actin-ADP-LatB (8BJH) and Vn-ExoY-3’dATP-2Mg^2+^:ATP-actin-LatB (8BR1). It appears that the structure of the free Pa-ExoY is designated as the first of two conformational states that is formed in the absence of co-factors.

What is the experimental evidence for a sequential mechanism for activation rather than a concerted or random mechanism? Conceivably, some of the conformational changes in switch regions and the lid domain could be independent and fluctuating, resulting in two stable end-point conformational states, rather than stable intermediates. It appears that the structure of the free Pa-ExoY is designated as the first of two conformational states that is formed in the absence of co-factors.

Do the authors have a hypothesis to explain why profilin enhances the affinity of Vn-ExoY for G actin, resulting in the actin assembly and AC catalytic rates shown in Figures 3B and 3C

The manuscript cites a number of other published structures of NC toxins that assume conformational states analogous to those for which structures are described for Vn-ExoY in the present manuscript. It would be of interest to this reviewer, and possibly to other readers with expertise in this subject, for the authors to provide a table listing the published structures of other NC Toxins that adopt conformations that are mechanistically similar or equivalent to the crystal structures presented in this manuscript.

Minor comments

Lines 139-145 and Fig 1 D. Kd values for inhibition of actin (+) end and (-) end assembly by Exo-Y are stated to be the same (~20 uM), yet the curvature of the two rate plots would suggest that the Kd for (-) end inhibition is about 2- fold lower. The latter also appears to have two phases.

Is there published literature that the reader could consult with regard to the derivation of the rate equations presented in the methods for Pyrene-actin polymerization assays?
