# Supplementary material for: Functional and structural insights into the multi-step activation and catalytic mechanism of bacterial ExoY nucleotidyl cyclase toxins bound to actin-profilin
Source: PLoS Pathog. 2023 Sep 25;19(9):e1011654. doi: 10.1371/journal.ppat.1011654 (PMC10553838; doi:10.1371/journal.ppat.1011654)
Supplement: Attachment — Submitted filename: 230729bPlosP-D2300717-3reviewersCONCERNSanswers.docx [file ppat.1011654.s021.docx]

Object: revision for manuscript PPATHOGENS-D-23-00716 -

**Friday, July 30, 2023**

Dear Editors,

We are sincerely grateful to the reviewers for their meticulous assessment and overwhelmingly positive feedback. In this revised manuscript, we have thoroughly addressed all the concerns and queries raised by the reviewers. Detailed responses to each concern are provided below.

We have improved and simplified the descriptions for readers who may not be familiar with the topic, eliminated somewhat redundant descriptions, and simplified several figures. Additionally, we have included the number of replicates in either figures or materials and methods sections. Moreover, we have integrated elements of the more detailed discussion into the captions of supplementary figures and tables, providing supplementary controls and information while reducing overall content.

Notably, we have significantly reduced and simplified the Discussion section to improve accessibility. Despite the inclusion of title subsections, additional explanations, and additional tables and figures in response to reviewer’s requests, the Discussion is now ~500 words shorter.

We are providing two versions of the revised manuscript: one with highlights or tracked changes, indicating the modified text, and the other as a clean version (uploaded as the manuscript file). Regarding data requirements, we have deposited all determined structures in the Protein Data Bank (PDB codes already included in the manuscript) and included an Excel file containing all numerical values used to generate the graphs in the manuscript. We also confirm that our reference list is complete and correct and includes the references used in both the main manuscript and all supplementary data.

With these extensive revisions, we firmly believe that the manuscript now aligns with the reviewers' expectations and meets the rigorous standards for publication in Plos Pathogen.

Sincerely yours,

L. Renault and all co-authors

**===== ===== Detailed responses to each concern ===== =====**

**Part II – Major Issues:**

**Reviewer #1:**

**The paper contains so many interesting data that it is almost unfair to ask for more. However, the length of the paper reduces its accessibility for non-experts.
I have the following suggestions for revision:**

**1. Please, add subsections to the Discussion.**

==authors==>

We have added the subsections. This should really help to clarify the discussion.

**2. Please, emphasize more clearly the novelty of your ExoY-3'dCTP structure.**

==authors==>

We have shortened and revised the results “**structural basis for the pyrimidinyl cyclase activity of ExoYs**“ and the Discussion to emphasize more clearly the novelty of our ExoY-3'dCTP structure in comparison to our previously reported actin-activated ExoY-3'dATP structures (with Mg^2+^ or Mn^2+^). In the Discussion, we have introduced a new subsection titled "**Key Elements for Purinyl and Pyrimidynyl Cyclase Activity of NC Toxin**s" where we underscore more specifically the significance of our findings.

In brief, our activated ExoY-3'dCTP structure, along with the corresponding structure with 3'dATP, unequivocally demonstrates that Vn-ExoY utilizes a two-metal-ion-assisted catalytic mechanism with both purine and pyrimidine nucleotides, as discussed. Our mutational analyses in Vn-ExoY and the distantly-related Pa-ExoY highlights shared features in the NC toxin active sites that likely play a crucial role in catalyzing the limiting steps of the cyclization reaction with substrates of varying sizes. This enhances the value of our findings in precisely understanding their catalytic mechanisms and specificities as potent Nucleotidyl Cyclases with broad substrate specificity. Furthermore, we have clarified in the Discussion**:**

“An essential feature allowing Vn-ExoY, and likely other NC toxins, to accommodate substrates of varying sizes in their NBP and to catalyse the cyclisation of large purine or small pyrimidine nucleotides is their remarkably similar and tight coordination with the nucleotide triphosphate moiety after closure of their C_B_/LID subdomain. However, in the structure of Vn-ExoY bound to actin and 3'dCTP-2Mg^2+^, the hydrogen bond network around the ribose and base is weaker than that observed for 3'dATP. This weaker interaction likely reduces the stability of pyrimidine nucleoside binding in the NBP of activated Vn-ExoY, which may explain why Vn-ExoY remains less efficient as CC than AC.”

3. **What about** **the Km, Vmax and Kcat values for all NTPs?**

==authors==>

These values have been determined solely for EF and CyaA. To consolidate some of the available data for class II NCs, we have introduced a new S1 table. Undoubtedly, obtaining essential biochemical characterizations for the actin-activated ExoY NCs is of utmost importance. We acknowledge this need and commit to addressing it in future research endeavors.

**4.** **What about** **potential compounds inhibiting ExoY?**

==authors==>

We have now added in the Discussion that the identification of potential inhibitors for the ExoY NC toxins has remained elusive to date. The new S1 Table presents available data on compounds identified and characterised against CaM-activated EF and CyaA NC toxins. Addressing this complex issue will be a key focus of future investigations. Our findings provide novel structural perspectives that can guide the development of additional strategies for inhibiting NC toxins.

**5. It would be most helpful to have a short summary table for the discussion comparing the most important features of ExoY, EF and CyaA.**

==authors==>

We have now included a new Table (Table S3) in the Discussion as requested. This table provides a concise comparison of the most significant interactions observed in the structures of actin-activated ExoY, CaM-activated EF, and CyaA in their nucleotide-free state or when bound to nucleotide substrate analogues or products resulting from the cyclization reaction. Notably, no previously solved structure exhibits as many contacts between a substrate nucleotide analogue (ATP or GTP) and the NC enzyme residues, two metal ions, and the NC residues, as well as between two metal ions and the nucleotide analogue. This comparison is further illustrated in detail in Figures 6A, 7A, and S7, where consistent thresholds are employed to analyze the different interactions with the nucleotide analogues and metal ions that act as cofactors in the cyclization reaction.

**Reviewer #2:**

**Discussion:** **a lot of the “Discussion” space is used to repeat the findings in the “Results” part**, **with multiple references to figures**. Without a doubt the authors can **improve the discussion by focusing on the important points, not repeating the results again and improving the accompanying illustration (Fig 8, see below).**

==authors==>

In response to the reviewers' feedback, we have made necessary improvements to the Discussion section, eliminating redundant descriptions and focusing on key points. Subsection titles have been added to provide clear guidelines. The length of the discussion has been significantly reduced by approximately 500 words while addressing additional information requested by the three referees. Non-redundant elements of the Discussion have been included in the captions of supplementary figures. To enhance clarity, we have improved and provided enlightening labels on the accompanying illustrations, including in Fig. 3, 4, 5, 6, 7 and 8.

**Fig. 4A-B. There is a lot going on here in each of the panels. I feel that the figure could be simplified by carefully selecting the most important things that the reader should look at and colouring those - reducing the complexity as much as possible, while keeping the message clear. Any solution could work as long as it reduces the impression of cacophony currently present in these two figure panels.**

==authors==>

We apologize for that and have simplified Fig. 4B as requested. Fig 4B shows now only the closed state of Vn-ExoY with 3’dATP, which can be compared to its open state of Vn-ExoY without nucleotide in Fig 4A.

**Fig. 4D. Similarly, it may be a good idea to reduce the complexity of the figure panel D. The fewer unnecessary elements are shown, the easier it is to follow what the authors are presenting.**

=>Authors=>

We have simplified Fig. 4B by removing many labels, keeping only the most important, which are used in the results or discussion.

**Fig. 6A** - **is it necessary to have such a complicated presentation in a main figure? Can this panel be simplified?** **I think it should be possible to have a panel that conveys the main ideas, but with fewer details shown all at once.**

=>Authors=>

We have indeed worked towards simplifying the figure while retaining all the essential information necessary for our analysis and conclusions. For improved clarity and simplicity, Vn-ExoY residue numbers are now displayed in all the manuscripts and figures without thousands. For example, V.n. MARTX residue 3412 is now represented as 412.

In our study, we extensively analyzed protein-ligand interactions within our structures and also referred to previously determined structures of class II NC toxins (EF, CyaA, or Pa-ExoY, S3 table, S7 fig), and class III AC, such as the one corresponding to PDB 1CJK (Fig 6F). This comprehensive analysis involved the use of reliable and consistent thresholds, established for analyzing various types of interactions between proteins and ligands, encompassing small molecules, ions, and water molecules.

By conducting stringent comparisons using the same thresholds and criteria for analyzing protein-ligand interactions, we can confidently conclude that the nucleotide-metal, nucleotide-NC enzyme, and metal-NC enzyme interactions are less tight and less competent for catalysis when compared to our structures with the closed LID and two bound metal ion cofactors.

As for the selection of only a subset of these interactions, we believe that making such a choice at this stage of knowledge, without computational studies on the most probable reaction sequence for catalysis of Vn-ExoY, would be too arbitrary. Instead, we present a simplified figure (S11 fig) containing an arbitrary subset of interactions, which were identified and proposed as crucial in a computational investigation of the tmAC's most probable catalysis mechanism. This investigation is based on the PDB 1CJK of tmAC, demonstrating similar interactions with the NBP of activated Vn-ExoY, binding 3’dATP and 2 metal ions.

**Fig. 6A and 6F.** **Although this is an interesting schematic representation of the nucleotide binding site comparison between the ExoY and the Gs-activated mAC, it would be more informative to show an example of the main residues in an active site of an actual mAC structure, compared with the active site in ExoY. It would be informative to see how active sites are organised in class II and class III cyclases, and how the new structures reported here advance our understanding of the similarities / differences.**

=>Authors=>

We appreciate the suggestion and have made efforts to simplify the figure while retaining all essential information necessary for our analysis and conclusions. In response, we propose an alternative representation in S11 figure. Fig. 6A and 6F offer a precise organization and synthesis of the coordination between the enzyme and ATP substrate analogue, the enzyme and metal ions, and the ATP substrate analogue and metal ions in the active site of actin-activated Vn-ExoY and Gs-activated mAC.

After exploring various schematic representations of the NC nucleotide binding sites, we found that these figures 6A and 6F provide the most straightforward illustration for thoroughly comparing the similarities and differences in the active sites of activated class II and class III cyclases. They effectively allow us to track and comprehend these similarities and differences, as described in the results, Discussion, and legend captions.

While three-dimensional representations are valuable, they quickly become overcrowded and challenging to compare between different cyclases due to corresponding residues occupying different orientations. However, we have included a simplified figure (S11 fig) that presents a three-dimensional representation for a minimal subset of interactions between the nucleotide and the class II and class III cyclases. This representation includes corresponding three-dimensional views side by side for better comparison.

Furthermore, to facilitate comparisons among the closest NCs of class II (EF, CyaA, and ExoYs), we have added Table S3. This table offers a quick and comprehensive comparison of key mechanistic and structural features of the known structures of the CaM-activated EF/CyaA and the actin-activated ExoY NC toxins.

**Fig. 8. The scheme is very complicated, and since there are no indices to follow, it is actually hard to decipher what is shown. The authors can try and optimise this figure by keeping a uniform style of presentation for different items shown in this figure (currently a rather eclectic mixture of different styles), and by using indices (such as “1, 2, 3, ..” or “A, B, C, ..”) to help convey the flow of what is shown and to guide the reader through the mechanism.**

==authors==>

We sincerely apologize for the oversight. The labels were inadvertently lost and overlooked during one of the recent updates of the figure. We have now addressed this issue by implementing a uniform style of presentation for different items, reintroducing the necessary indices on the figure, and improving the legend and discussion to facilitate a clear understanding of the various model steps related to the activation and activity of Vn-ExoY.

**Reviewer #3:** Major comments

**These conclusions are supported by the structural, mutagenic and actin-assembly and Cyclase assays.**

**1-However, while error bars are shown for some of the assays (e.g., Figure 5) there is no discussion of the number of technical or “biological” replicates used in the various assays or methods used, if any, to validate the statistical significance of measured or derived rate constants presented in the manuscript.**

==authors==>

We sincerely apologize for this oversight and have now rectified it. We have added the number of replicates used in the various assays for Figure 1, 2, 3-B-C, 5A, 5C, 6C-D, 7B-C, S1, and S2 Fig. This information has been included in the figure legends or in the Materials and Methods section.

**2-What is the experimental evidence for a sequential mechanism for activation rather than a concerted or random mechanism? Conceivably, some of the conformational changes in switch regions and the lid domain could be independent and fluctuating, resulting in two stable end-point conformational states, rather than stable intermediates. It appears that the structure of the free Pa-ExoY is designated as the first of two conformational states that is formed in the absence of co-factors.**

==authors==>

The experimental evidence for a sequential mechanism of activation, as opposed to a random mechanism, lies in the observation that free NC toxins exhibit extremely weak basal activities, rendering them nearly inactive without their respective cofactors. For instance, the background activity of free Vn-ExoY in the absence of actin is approximately 0.01 μmol/min/mg (Ref. [5]). To further demonstrate this, we have included the fold stimulation of their NC activity by their corresponding cofactor in the new Table S1, and in the Discussion that G-actin:profilin acts as a potent cofactor for Vn-ExoY, significantly enhancing its basal AC activity (Ref. [5]) by over 30,000-fold at a concentration of only 7 µM (375 μmol/min/mg from Fig 2A).

As summarized in the Discussion and depicted in Fig. 6A, 7A, and S7, the nucleotide binding sites in NC toxins rely on four distinct regions: subdomain C_A_, C_B_/LID, switch A, and switch B. Molecular dynamics simulations on CyaA (ref.[52]) and Pa-ExoY (ref.[28]) indicate that the C_B_/LID, switch A, and switch B regions display inherent flexibility and fluctuations when the cofactor is absent. In the case of EF, the presence of a C-terminal extra-helical domain stabilizes switch A and switch B, a feature unique to EF. In the crystal structure of free EF, the conformations of switch A and switch B are incompatible with CaM binding, necessitating significant rearrangements upon cofactor binding (ref. [24]). Consequently, they adopt a distinct conformation that promotes catalysis by approaching switch B and the NBP. Interpreting the crystal structure of free Pa-ExoY obtained after limited in-situ proteolysis (ref. [26]) proves more challenging. The three switches are mostly indiscernible but the precise contribution of partial proteolysis and/or inherent flexibility to this observation remains unclear. In the subsequent steps, crystal structures of CaM-bound EF without ligand reveal that the C_B_/LID can occupy different positions and therefore undergo fluctuations. To facilitate a comprehensive comparison of known structures of NC toxins, we have included the new Table S3, which illustrates these features in a clear and concise manner.

Our structures complement and further elucidate these findings. As summarised in the Discussion, in the absence of cofactor, the substrate-induced closure of the NBP fails to catalyse the reaction effectively, as switch A and B, and hence, the coordination of Mg^2+^_A_ and the substrate nucleoside moiety (Fig 6A, 7A), remain improperly stabilised. Our structures of Vn-ExoY bound to actin:profilin without a nucleotide (PDB 8BJH, 8BJI, 8BJJ) reveal that switch A remains partially disordered, while switch B displays high B-factors compared to its actin-bound structures with 3'dATP or 3'dCTP (Fig. 4D). Moreover, our determined structures of actin-bound Vn-ExoY with or without substrate analogues (Fig 4A-B) and previously determined structures of CaM-bound EF with or without substrate analogues (Fig 6E, S8, and S3 table) demonstrate fluctuations in the CB/LID region in the presence of cofactors. Additionally, our data demonstrate that NC toxins employs a two-metal-ion-assisted catalytic mechanism with purine and pyrimidine nucleotides. Regarding that, our Vn-ExoY:actin structures without nucleotides and the crystal structure of free Pa-ExoY show that part of the full coordination of Mg^2+^_A_ is not available. As we underline in the Discussion: “in both the inactive conformation of free Pa-ExoY [26] and the nucleotide-free conformations of actin:profilin-bound Vn-ExoY (Fig 3D-E), the metal-binding H291^PaE-switch-B^ and H753^VnE-switch-B^, respectively, are stabilised away from the NBP by a salt bridge with D736^VnE^/D274^PaE^, positioned near switch A (Fig 4D). Upon binding both the cofactor and the substrate, ExoYs exhibit a flipping motion of the H753^VnE-switch-B^/H291^PaE-switch-B^ towards the NBP. This invariant histidine in NC toxin switch B significantly contributes to the precise positioning of the catalytic Mg^2+^_A_ near the two invariant aspartates (D665D667^VnE-CA^ or D212D214^PaE-CA^) with purine or pyrimidine nucleotide substrate analogues (Fig 6A-B, 7A).”

Taken together, these observations strongly favor a sequential mechanism of activation, providing valuable insights into the intricate interplay between NC toxins, cofactors, and substrates. Further investigations will undoubtedly enhance our understanding of these mechanisms.

**3-Do the authors have a hypothesis to explain why profilin enhances the affinity of Vn-ExoY for G actin, resulting in the actin assembly and AC catalytic rates shown in Figures 3B and 3C**

==authors==>

The observed increase in the affinity of Vn-ExoY for G-actin shown in Figures 3B and 3C can be attributed to the specific arrangement of the proteins in the chimera or fusion construct. In these figures with the chimera, Vn-ExoY is covalently fused to the N-terminus of profilin, with an additional Profilin-binding Proline-Rich Motif (PRM) in between (Fig. 3A). As a result, this fusion protein contains both Vn-ExoY and profilin, which can bind to G-actin simultaneously without disturbance or competition between each other. Consequently, the binding of both Vn-ExoY and profilin to G-actin synergizes, leading to a 100-fold increase in the affinity for G-actin compared to the isolated, wild-type Vn-ExoY (Fig. 3C). We have added these supplementary explanations in the materials and methods section. Additionally, in the Discussion and new Table S1, we have presented that a G-actin:profilin complex at 7 µM concentration can stimulate the basal Adenylyl Cyclase activity of Vn-ExoY by more than 30,000-fold (as previously measured in ref. [5]). This level of stimulation is comparable to the strong stimulation observed for Pa-ExoY by F-actin (S1 Table).

4-**The manuscript cites a number of other published structures of NC toxins that assume conformational states analogous to those for which structures are described for Vn-ExoY in the present manuscript. It would be of interest to this reviewer, and possibly to other readers with expertise in this subject, for the authors to provide a table listing the published structures of other NC Toxins that adopt conformations that are mechanistically similar or equivalent to the crystal structures presented in this manuscript.**

==authors==>

In response to the reviewer's request, we have added a new Table S3 to our revised manuscript. This table provides a comprehensive list of published structures of other NC toxins that interact with their respective cofactors, with or without ligands. Notably, the table indicates that there are currently no published structures of cofactor-bound NC toxins with a nucleotide substrate analogue of ATP/GTP in their Nucleotide-Binding Pocket (NBP) that exhibit coordination and conformations mechanistically similar or equivalent to the crystal structures of actin-activated Vn-ExoY utilizing 3'dATP or 3'dCTP as bound nucleotide substrate analogues with two metal ions (presented in our manuscript).

**Part III – Minor Issues: Editorial and Data Presentation Modifications**

Please use this section for editorial suggestions as well as relatively minor modifications of existing data that would enhance clarity.

**Reviewer #1:** (No Response)

**Reviewer #2:** **Supporting Information: please add protein biochemistry data SDS PAGE, gel filtration profiles, etc. Although it is clear that the X-ray structures have been determined at high resolution, it makes sense to see the purity of the samples used in functional assays.**

==authors==>

We have included Figure S12, which displays representative Coomassie blue stained SDS-PAGE (15%) gel images, assessing the purity of the samples utilized in the functional assays. Additionally, Figure S13 exhibits gel filtration profiles for the Vn-ExoY and Pa-ExoY constructs employed in the NC activity assays.

The functionality of our protein constructs has been substantiated through actin binding assays, actin-dependent NC activity assays, and high-resolution structures. These comprehensive analyses demonstrate the effectiveness and reliability of our protein constructs.

**Fig. 1-3.** **How many times were the experiments repeated? Is n in Fig. 2B a number of replicates or repeats? Number of repeats should be specified for each figure panel where experimental data is presented.**

==authors==>

We sincerely apologize for this oversight, as already mentioned by reviewer 3, and we have addressed it accordingly: We have added the number of replicates used in the various assays for Figure 1, 2, 3-B-C, 5A, 5C, 6C-D, 7B-C, S1, and S2 Fig. This information has been included in the figure legends or in the Materials and Methods section. Regarding Figures 1E and 2B, the c(s) distribution represents a single experiment that is representative of at least two independent experiments. These standard procedures for analytical ultracentrifugation analyses ensure the validity and reliability of the data presented.

**Fig. 3C.** **Growth rates are plotted against the toxin concentration, but “Kd” values are shown. What does this mean? What is “Kd” in this case? Although it is clear that Kd should be the dissociation constant in a binding assay, the meaning of Kd here is less clear.**

==authors==>

We confirm that in Fig 3C and 1D, the fit corresponds to a dissociation constant between the Vn-ExoY protein constructs (as Actin-binding Protein, ABP) and G-actin. In this context, Kd represents the equilibrium dissociation constant for the ABP:actin complex. This value is derived from the dependence of the elongation rate at (+) or (-) ends on the concentration of the ABP.

To provide clarity, we have made the necessary modifications to the equation. Specifically, we have changed "Kc" to "K_D_" in eq.2, and we now specify that in eq.2, K_D_ represents the equilibrium dissociation constant for the binding between the actin-binding protein (C) and G-actin (A).

We trust that this clarification will enhance the understanding of our findings and facilitate interpretation for readers.

**Fig. 4D.** **In the transition from D-1 to D-2 - is that ATP molecule above the arrow? Or an ATP analogue?**

==authors==>

The label for 3'dATP, the ATP analogue bound to two metal ions in the closed conformation of our actin-bound Vn-ExoY structure, has been added to the figure.

**Fig. S2.** **How many times were these experiments performed?**

==authors==>

We have added the missing information in the figure legend: "Single experiments of actin polymerisation kinetics are shown. They are representative of a minimum of 3 independent experiments".

**Fig. S3C.** **There might be a problem with the labels.**

==authors==>

We apologize for this mistake left and have corrected the labels that were only half visible.

**Fig. S6.** **The overlayed structures are very crowded - as a result the active site heterogeneity does not immediately come across. In fact, the active site itself appears to not be all that heterogeneous.**

==authors==>

We have addressed the issue of crowding in Fig. S6 by simplifying the presentation. Now, only two structures are shown overlaid at once, and these overlays are displayed two by two in multiple panels to provide a clearer demonstration of the active site heterogeneity between NC toxins, nucleotide substrate analogues, and 1 or 2 metal ions.

In addition, Fig. S7 complements Fig. S6, offering together unambiguous evidence of the variations in the number of ions, the position and coordination of metal ion(s), and the position, conformation, and coordination of nucleotide substrate analogues. This includes variations in the coordination between the enzyme and nucleotide, as well as between the nucleotide and the metal ion(s). For instance, panel S6-B illustrates that the adenine ring of the AMPCPP bound to CaM-bound EF (PDB 1S26) is rotated approximately 180° compared to 3’dATP bound to CaM-bound EF (PDB 1XFV, 1K90) concerning the nucleotide conformation. Moreover, panels S6-A and S6-C demonstrate that 3’dATP (S6-A Fig) or 3’dGTP (S6-B Fig) can be bound with either 1 or 2 metal ions in previous structures. Additionally, the conserved K353EF-LID (S6-A Fig) or K88PaE-LID (S6-C Fig), equivalent to K535^VnE-LID^ in Vn-ExoY (Fig 6A, 7A), can either be away from the γ-phosphate oxygens or coordinate with them.

**Fig. S8-S9** - the **resolution of the** **figure can be improved.**

==authors==>

The figures are shown with a higher resolution of at least 300 DPI.

**Reviewer #3:**

Minor comments

**Lines 139-145 and Fig 1 D. Kd values for inhibition of actin (+) end and (-) end assembly by Exo-Y are stated to be the same (~20 uM), yet the curvature of the two rate plots would suggest that the Kd for (-) end inhibition is about 2- fold lower. The latter also appears to have two phases.**

==authors==>

Equation 1 (eq. 1) describes the G-actin sequestration activity of an actin-binding protein (ABP) based on the concentration dependence of rates of barbed and pointed end growth at a given concentration of G-actin. It is important to note that the critical concentrations (Cc) at the (+) end and (-) end assembly differ, with values of 0.1 µM and 0.6 µM, respectively. This disparity in the Cc values within eq. 1 leads to distinct curves representing the formation of a sequestering complex (by its Kd value) as a function of the ABP concentration. Therefore, despite the observed differences in the curves, the same Kd value of ~20 µM fits both independent experiments investigating the concentration-dependence inhibition of (+) and (-) end growth by Exo-Y.

**Is there published literature that the reader could consult with regard to the derivation of the rate equations presented in the methods for Pyrene-actin polymerization assays?**

==authors==>

We have now incorporated two additional references in the materials and methods section, elucidating the previously derived equations for calculating the equilibrium dissociation constant (Kd) based on the ABP's concentration dependence of rates of barbed and pointed end growth. By integrating these elements with the equations provided in the manuscripts, all the equations can be derived.
